# Boosting CO hydrogenation towards C_2+_ hydrocarbons over interfacial TiO_2−*x*_/Ni catalysts

**DOI:** 10.1038/s41467-022-34463-7

**Published:** 2022-11-07

**Authors:** Ming Xu, Xuetao Qin, Yao Xu, Xiaochen Zhang, Lirong Zheng, Jin-Xun Liu, Meng Wang, Xi Liu, Ding Ma

**Affiliations:** 1 College of Chemistry and Molecular Engineering, Peking University, Beijing, 100871 P. R. China; 2 State Key Laboratory of Chemical Resource Engineering, Beijing University of Chemical Technology, Beijing, 100029 P. R. China; 3 Institute of High Energy Physics, the Chinese Academy of Sciences, Beijing, 100049 P. R. China; 4 Department of Chemical Physics, University of Science and Technology of China, Hefei, 230026 P. R. China; 5 School of Chemistry and Chemical Engineering, In-situ Center for Physical Sciences, Shanghai Jiaotong University, Shanghai, 200240 P. R. China; 6 Syncat@Beijing, Synfuels China Co., Ltd, Beijing, 101400 P. R. China

**Keywords:** Heterogeneous catalysis, Chemical engineering, Catalytic mechanisms

## Abstract

Considerable attention has been drawn to tune the geometric and electronic structure of interfacial catalysts *via* modulating strong metal-support interactions (SMSI). Herein, we report the construction of a series of TiO_2−*x*_/Ni catalysts, where disordered TiO_2−*x*_ overlayers immobilized onto the surface of Ni nanoparticles (~20 nm) are successfully engineered with SMSI effect. The optimal TiO_2−*x*_/Ni catalyst shows a CO conversion of ~19.8% in Fischer**–**Tropsch synthesis (FTS) process under atmospheric pressure at 220 °C. More importantly, ~64.6% of the product is C_2+_ paraffins, which is in sharp contrast to the result of the conventional Ni catalyst with the main product being methane. A combination study of advanced electron microscopy, multiple in-situ spectroscopic characterizations, and density functional theory calculations indicates the presence of Ni^*δ−*^/TiO_2−*x*_ interfacial sites, which could bind carbon atom strongly, inhibit methane formation and facilitate the C-C chain propagation, lead to the production of C_2+_ hydrocarbon on Ni surface.

## Abstract (web summary)

Considerable attention has been drawn to tune the geometric and electronic structure of interfacial catalysts *via* modulating strong metal-support interactions (SMSI). Here the authors report the remarkable catalytic performance of CO hydrogenation over an interfacial TiO_2−*x*_/Ni catalyst by means of SMSI.

## Introduction

CO hydrogenation to high value-added chemicals *via* Fischer**–**Tropsch synthesis (FTS) process has attracted growly attention, which has become the most frontier field in academia and industry^1–6^. Despite of great success in industry, there is still much debate over reaction mechanism and governing structure-activity relationship, which mainly originates from extensive studies on Fe or Co-based FTS. It is of vital importance to find an alternative to Co or Fe catalysts and thereafter develop mechanistic understanding of structure-activity relationship from a new perspective^7–11^. Ni-based catalysts had been considered as a vital candidate for FTS for a long time as they displayed high activities for CO hydrogenation under relatively mild operation conditions compared to those in Co or Fe-catalyzing FTS, prevailing methanation and fast deactivation due to the formation of Ni-carbonyl severely hinder their practical applications^12–14^. However, some experiments and calculations reported the metal-support interactions and its effect on product distributions in Ni-catalyzing FTS^15,16^, which suggests that the optimal Ni-based catalysts may display similar FTS activity to Co catalysts^17^. Therefore, it is of great interest to develop a new type of Ni-based catalyst to efficiently inhibit the formation of nickel carbonyl and simultaneously enhance the C–C chain propagation, so as to increase the selectivity of CO hydrogenation towards high hydrocarbons. As indicated by the aforementioned works^18–21^, modulating the geometric and electronic structures of Ni particles should be an effective way to tune the catalytic performance of CO hydrogenation towards long-chain hydrocarbons.

Strong metal-support interaction (SMSI) was firstly put forward by Tausters et al. in 1978^22,23^, which alters the geometric and electronic structures of supported metal particles, especially for the interfacial properties^24^. Suboxides migrated on the surface of supported metal particles with strong electron transfers at the interface is a typical characteristic of SMSI, resulting in the re-arrangement of atoms or charge redistribution of the interface^25–28^. Actually, many SMSI-type catalysts have been developed to substantially enhance the catalytic activity and selectivity of CO hydrogenation towards desirable long-chain hydrocarbons in C1 chemistry^29,30^. De Jong et al. designed a reduction-oxidation-reduction (ROR)-SMSI type Co-based catalyst with high exposure of active interfacial sites and metal dispersion to efficiently increase the catalytic performance of FTS reaction^31^. In addition, the catalytic properties of Ru/TiO_2_ catalysts could be also efficiently improved *via* SMSI during FTS, owing to promoting the activation and cleavage of CO bond by TiO_2−*x*_ overlayers located at Ru/TiO_2_ interface^32^. Compared to traditional impregnation method, in situ structural transformation of Layered double hydroxides (LDHs) could provide an efficient cutting-edge approach to construct new-type SMSI catalyst, which has been explored in C1 catalytic chemistry in recent years^33,34^. These results inspire us to synthesize a new-type Ni-based SMSI catalyst with enhanced FTS performance with respect to its academic and practical significance.

In this work, SMSI-type TiO_2−*x*_ overlayers decorated Ni nanoparticle catalysts (TiO_2−*x*_/Ni) were successfully synthesized *via* the reduction of calcinated ultrathin NiTi-LDHs precursor. Abundant Ni^*δ−*^/TiO_2−*x*_ interfacial sites can be found on TiO_2−*x*_/Ni catalyst, which were validated by quasi in situ XPS, in situ EXAFS, and in situ DRIFTS in detail. The optimal TiO_2−*x*_/Ni catalyst exhibits a CO conversion of ~19.8% with ~64.6% selectivity to C_2+_ paraffin at 220 °C under atmosphere pressure. Both experimental and theoretical results evidence that the Ni^*δ−*^/TiO_2−*x*_ interfacial sites at TiO_2−*x*_/Ni catalysts can facilitate CO activation and C-C chain propagation to produce C_2+_ paraffin but hinder methane formation. The well-designed LDHs transformation is proved to be an efficient approach to construct the new-type SMSI catalysts with the controllable exposure of abundant active interfacial sites, which paves a new way to the rational design of high-performance heterogeneous catalysts.

## Results

### Synthesis of TiO_2−*x*_ overlayers decorated Ni nanoparticles (TiO_2−*x*_/Ni) catalysts

**Fig. 1:**
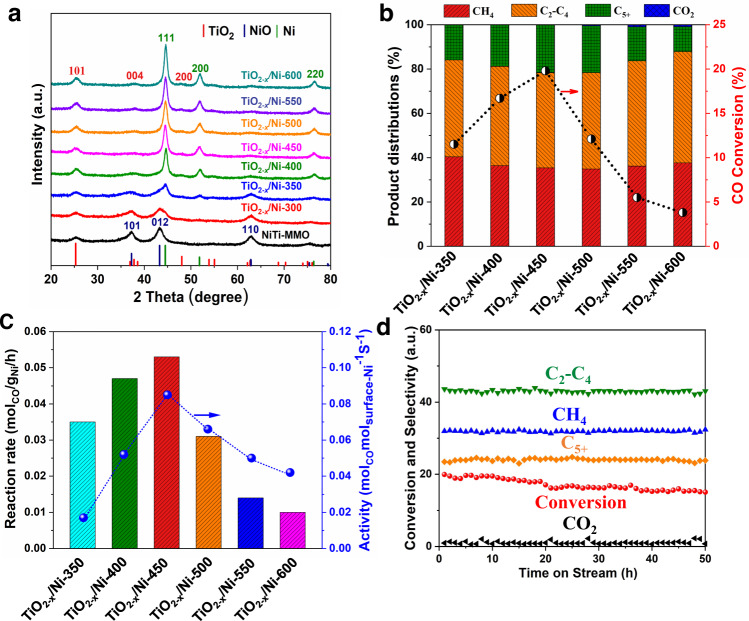
Structural properties and catalytic performance of TiO_2−*x*_/Ni catalysts in syngas conversion under different conditions. **a** XRD patterns of NiTi-MMO and a series of TiO_2−*x*_/Ni catalysts reduced at different temperatures. **b** The conversion and product distribution as well as (**c**) reaction rate and activity over various TiO_2−*x*_/Ni catalysts. **d** The stability evaluation of TiO_2−*x*_/Ni-450 at 220 °C. Reaction conditions: catalysts (120 mg), 1 bar, syngas (CO/H_2_/Ar = 32/64/4; space velocity: 10000 mL g_cat_^−1^ h^−1^).

The NiTi-LDHs nanosheets were successfully synthesized by the hydrothermal synthesis approach described in the previous work^35^. The XRD pattern shows a series of diffraction peaks at 2*θ* of 12.0°, 24.6°, 33.4°, 37.9°, and 59.8°, which are corresponded to the (003), (006), (009), (012), and (110) planes of NiTi-LDHs phase (Supplementary Fig. 1a)^36^. The as-synthesized NiTi-LDHs nanoplates display a mean diameter of ~200 nm with an average thickness of ~6 nm (Supplementary Fig. 1b–d). After calcination at 500 °C for 4 h, mixed oxides (NiTi-MMO) composed of NiO and TiO_2−*x*_ are obtained (Supplementary Fig. 2). The primary diffraction peaks at 37.3°, 43.5°, 63.2° can be ascribed to the cubic NiO crystal phase^37^ (Supplementary Fig. 2a). The anatase phase of TiO_2−*x*_ with weak and broad reflection could be observed with respect to its poor crystal structure. As seen in the TEM image (Supplementary Fig. 2b–d), small NiO nanoparticles are well-dispersed into the oxide composite. Afterwards, the oxide mixture (NiO and TiO_2*-x*_) was reduced by H_2_ at 300 °C, 350 °C, 400 °C, 450 °C, 500 °C, 550 °C, and 600 °C, respectively. The reduced samples are designated as TiO_2−*x*_/Ni-*T*, with *T* denoting the reduction temperature. Inductively coupled plasma atomic emission spectrometry (ICP-AES) measurements confirm around 50 wt% of nickel loadings in the reduced samples (Supplementary Table 1). Notably, formation of metallic nickel could be observed after reduction at 350 °C according to the XRD data (Fig. 1a). By increasing the reduction temperature, the XRD signals was getting increased and narrower, it indicates the growth of Ni particles when raising up the temperature (Fig. 1). According to the Scherrer equation, the particle sizes increase from **~**15 nm to **~**20 nm within the temperature raising from 400 °C to 600 °C (Supplementary Table 1). TEM was carried out to further explore the metal dispersion (Supplementary Fig. 3 and Supplementary Table 1), which agrees well with the XRD characterizations.

### Evaluation of catalytic performance

The catalytic performances of TiO_2−*x*_/Ni catalysts reduced at different temperatures were evaluated at 220 °C with a weight hourly space velocity (WHSV) of 10,000 ml g_cat_^−1^ h^−1^, as shown in Fig. 1b and Supplementary Table 2. Promisingly, the product distributions demonstrate that C_2+_ paraffin is a predominant product rather than methane (CH_4_) for CO hydrogenation over TiO_2−*x*_/Ni catalysts. The six TiO_2−*x*_/Ni-T catalysts show similar C_2+_ paraffin selectivity at the same conditions, indicating all the catalysts contain similar active sites. CO conversion increases initially but decreases subsequently when the catalyst activation temperature increases from 350 to 600 °C, suggesting that the decrease of active sites during reduction at higher temperature. The optimal TiO_2−*x*_/Ni-450 catalyst exhibits the highest CO conversion (~19.8%) at 220 °C under atmosphere pressure with the selectivity of C_2+_ paraffin up to ~64.6% (Supplementary Fig. 4). The selectivity of C_5+_ products can attain ~21.5% at the relatively mild conditions. When switching to a lower reaction temperature of 200 °C, CO conversion of the optimum TiO_2−*x*_/Ni-450 catalyst is decreased to ~6.8% associated with a higher selectivity of C_2+_ paraffin (~72.3%, Supplementary Fig. 5 and Supplementary Table 3). Obviously, the activity and selectivity of CO hydrogenation are significantly dependent on the reaction temperature. The catalytic reaction rates and activities of various TiO_2−*x*_/Ni catalysts show a volcano-like trend with the increment of catalyst reduction temperature in Fig. 1c. The optimum TiO_2−*x*_/Ni-450 catalyst shows the highest reaction rate (0.053 mol_CO_ g_Ni_^−1^ h^−1^) and catalytic activity (0.085 mol_CO_ mol_suface-Ni_^−1^ s^−1^) at 220 °C. Additionally, TiO_2−*x*_/Ni-450 catalyst displays a good stability (20% activity decrement) within 50 h at 220 °C (Fig. 1d).

As a comparison, the 15% Ni/SiO_2_ catalyst (Supplementary Figs. 6 and 7) was also evaluated. However, the 15% Ni/SiO_2_ exhibit a poor CO conversion (2.4%) and much higher selectivity of CH_4_ (67.9%) with a reaction rate (0.031 mol_CO_ g_Ni_^−1^ h^−1^) and catalytic activity (0.007 mol_CO_ mol_suface-Ni_^−1^ s^−1^) at the same condition. Obviously, the selectivity of C_5+_ paraffin for TiO_2−*x*_/Ni-450 is ~21.5%, which is much higher than that of 15% Ni/SiO_2_ (C_5+_ paraffin selectivity: 1.4%) in Supplementary Fig. 8 and Supplementary Table 4. In addition, it is worth noting that the catalytic activity of TiO_2−*x*_/Ni-450 is ~12-fold than that of Ni/SiO_2_. When the Ni loading increases to 30 wt% (Supplementary Fig. 9), the 30% Ni/SiO_2_ catalyst exhibits an even worse CO conversion (1.0%) and much higher selectivity of CH_4_ (80.3%). The catalytic activity of 30% Ni/SiO_2_ is lower than that of 15% Ni/SiO_2_ at 220 °C with higher selectivity of CH_4_. It suggests that there is no SMSI in the SiO_2_ supported catalysts, or the SMSI effect present in the Ni/SiO_2_ catalyst system is significantly different from that in the TiO_2−*x*_/Ni-450 system. In order to explore the influence of different support, catalytic performance of the conventional Ni/TiO_2_ prepared by a conventional impregnation method was also examined (Supplementary Fig. 10). Clearly, the catalyst displays much worse catalytic performance at 220 °C (10.1% conversion and 7.3% selectivity to C_5+_ paraffin) in contrast to TiO_2−*x*_/Ni-450 (19.8% conversion and 21.5% selectivity to C_5+_ paraffin). Therefore, the support effect between Ni and TiO_2_ play an important role in improving the catalytic activity and selectivity towards to C_2+_ paraffin.

In order to understand the active sites, the apparent activation barrier (*E*_a_) of CO hydrogenation was measured over various TiO_2−*x*_/Ni catalysts in Supplementary Fig. 11. The kinetic studies show that apparent activation barrier of CO hydrogenation is similar over various TiO_2−*x*_/Ni catalysts, i.e., the intrinsic active sites is similar, resulting the similar product selectivity. It is obvious that the catalytic activity first raises and then declines even when normalized per surface Ni as shown in Supplementary Fig. 12. This is attributed to the surface Ni concentration decreases with the increment of reduction temperature, owing to the influence by the SMSI effect at the high temperature, i.e., the sub-oxide (TiO_2−*x*_) migrated gradually on the surface of metal Ni particles driven by SMSI. Simultaneously, the active interfacial Ni^*δ−*^/TiO_2−*x*_ sites decrease gradually with the increment of reduction temperature after 450 °C. This indirectly indicates that the interfacial Ni^*δ−*^/TiO_2−*x*_ sites were a key factor governing the catalytic efficiency whilist the surface Ni concentration.

### The identification of Ni^*δ−*^/TiO_2−*x*_ interfacial sites over various TiO_2−*x*_/Ni catalysts

**Fig. 2:**
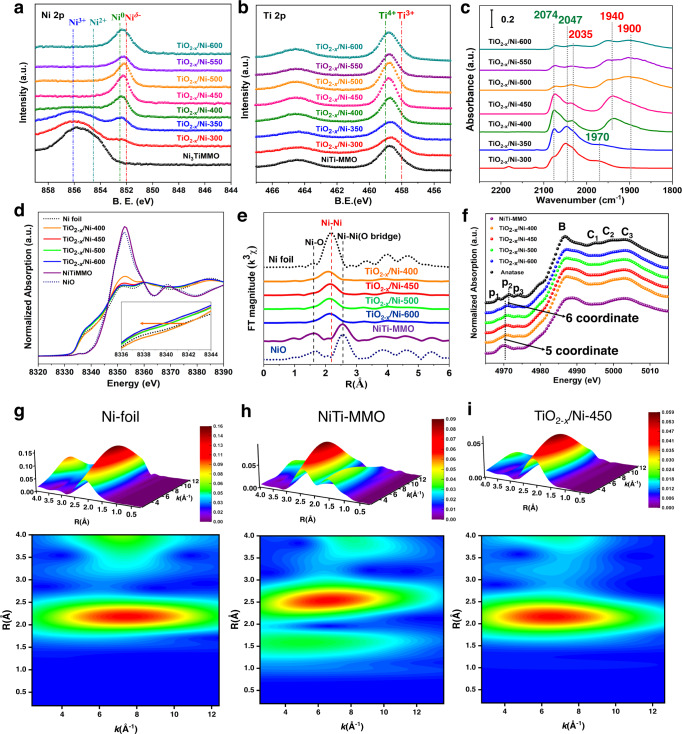
The revelation of geometric and electronic structure for various TiO_2−*x*_/Ni catalysts. Quasi in situ XPS spectra of (**a**) Ni 2*p* and (**b**) Ti 2*p* for various TiO_2−*x*_/Ni catalysts. **c** In situ CO-DRIFTS spectra of TiO_2−*x*_/Ni catalysts at room temperature. **d** The normalized Ni *K*-edge XANES spectra of various TiO_2−*x*_/Ni catalysts, respectively. **e** The corresponding normalized Ni *K*-edge Fourier-transform EXAFS spectra. **f** The Normalized Ti *K*-edge XANES spectra of various TiO_2−*x*_/Ni catalysts. WT analysis of (**g**) Ni foil, (**h**) NiTi-MMO, and (**i**) TiO_2−*x*_/Ni-450 catalyst.

Quasi in-situ XPS was carried out to reveal the surface electronic structures of various TiO_2−*x*_/Ni samples activated at different temperatures (Fig. 2a, b). For NiTi-MMO^11,38^ prepared *via* the calcination of NiTi-LDHs precursor, a 2*p*_3/2_ peak at 853.4 eV is assigned to Ni^2+^ species^39,40^ and a peak at ∼856.1 eV is indexed to Ni^3+^ species due to the formation of abundant V_Ni_ species in NiTi-MMO^37,41^, both of which demonstrate that Ni^3+^ and Ni^2+^ species exist in NiTi-MMO. Whereas a weak 2*p*_3/2_ peak at 852.5 eV can be observed in TiO_2−*x*_/Ni-300 catalyst, which is indexed to Ni^0^ species^42^. With the reduction temperature raising up to 400 °C, a stronger 2*p*_3/2_ peak at 852.0 eV can be found and attributed to the Ni^*δ−*^ species^17,21^. When the reduction temperature rises to 600 °C, the 2*p*_3/2_ peak located at 852.0 eV becomes broader and stronger, indicating a higher content of Ni^*δ−*^ species in the further reduced catalyst. The corresponding amount of Ni^*δ−*^ species could be quantified over various TiO_2−*x*_/Ni catalysts with a deconvolution by Gaussian peak fitting method (Supplementary Fig. 13 and Supplementary Table 5). According to Ti 2*p* XPS spectra, presence of Ti^3+^ species^43,44^ can be observed at 458.0 eV over various TiO_2−*x*_/Ni catalysts in Fig. 2b. It is very imperative to monitor the structure change of TiO_2−*x*_/Ni catalysts during the reaction, so as to confirm the stability of catalysts under the realistic reaction condition. It is obviously that the electronic structure of metal Ni species for TiO_2−*x*_/Ni-450 catalyst unchanged under the reaction condition at 220 °C as shown in Supplementary Fig. 14.

To further confirm the presence of electron-enriched Ni^*δ−*^ species over various TiO_2−*x*/_Ni catalysts, in situ CO-DRIFTS chemisorption experiments were carried out (Fig. 2c). The IR bands at ~2074, ~2047, and ~2035 cm^*−*1^ at 25 °C can be assigned to linear CO molecules on Ni defect sites, Ni^0^ atop sites, and Ni^*δ−*^ atop sites, respectively^45,46^. With the temperature rising up from 350 °C to 600 °C, the bands decrease owing to better crystallinity and coverage of TiO_2−*x*_ suboxides, thereafter inhibiting the formation of nickel carbonyl^17^. It is well known CO adsorption can be strongly modulated by the electronic structure of supported metals. Therefore, the electron-enriched Ni^*δ−*^ species with much more *d*-electron can enhance the back-donation into 2π* antibonding orbital of CO molecules^21^, and then strengthen CO chemisorption. It is observed that, when the temperature treatment increased from 300 °C to 450 °C, the band at ~1970 cm^*−*1^ ascribed to bridged CO adsorbed on Ni^0^ sites shifted to ~1940 cm^*−*1^ accompanied with the formation of three-fold carbonyl C = O stretching vibrations at ~1900 cm^*−*1^, it is due to the increment of Ni^*δ−*^ species which bonds the CO molecules stronger than Ni^0^ ^45^. According to our previous work^47^, the linear and bridge adsorbed CO can desorb from the surface of Ni^0^ sites after flow in the Ar atmosphere for 30 min above elevated temperatures. Whilst the linear and bridge chemisorbed CO species on Ni^*δ−*^ sites are even stable at 250 °C on TiO_2−*x*/_Ni-450 after Ar flow for 30 min (Supplementary Fig. 15). It further confirms the presence of significant amounts of Ni^*δ−*^ sites with a higher affinity for CO.

In situ XAFS spectra were obtained to reveal the electronic state, geometric structure, and coordination environment of various TiO_2−*x*_/Ni samples. In terms of NiTi-MMO, the normalized Ni *K*-edge XANES (Fig. 2d) displays a higher white line and the adsorption edge shifts toward the higher photon energy compared to the NiO reference, indicating that Ni^3+^ species present in the NiTi-MMO precursor in accordance with the result of XPS spectra. After the precursor was activated at 400 °C in 10% H_2_/He atmosphere at 400 °C, the XANES spectrum of TiO_2−*x*_/Ni-400 exhibits a slightly stronger white line compared to other TiO_2−*x*_/Ni catalysts, but is very close to that of Ni foil, further demonstrating that the predominant phase is the metallic nickel with a slight oxidation, i.e., the co-existence of Ni metal and NiO phase. Within the reduction temperature varying from 450 to 600 °C, the adsorption edge displays a shift toward lower energy than that for Ni foil and the white line is close to the Ni foil. Combined with the XPS and CO-DRIFTS characterizations, we can further confirm the formation of electron-enriched Ni^*δ−*^ species over the TiO_2−*x*_/Ni-400, TiO_2−*x*_/Ni-450, TiO_2−*x*_/Ni-500, and TiO_2−*x*_/Ni-600 catalysts. The electron-enriched Ni^*δ−*^ species is due to the strong electron donation from TiO_2−*x*_ to the interfacial Ni atoms by SMSI. In the case of R space plot for TiO_2−*x*_/Ni within the reduction temperature varying from 400 to 600 °C in Fig. 2e, the Ni-Ni shell of the four catalysts shows the similar coordination number in Supplementary Fig. 16, indicating they have similar particle size in line with the result of XRD and HRTEM images. The coordination number, bond distance, Debye-Waller factor, and other parameters determined by EXAFS characterizations are listed in the Supplementary Table 6. Wavelet transform (WT) of Ni *K*-edge EXAFS oscillations of Ni foil (Fig. 2g), NiTi-MMO (Fig. 2h) and TiO_2−*x*_/Ni-450 (Fig. 2i) indicate that NiTi-MMO was reduced to metallic particles. A prominent peak can be observed at 2.2 Å for Ni particles, which can be ascribed to the Ni-Ni scattering. The similar phenomenon can be observed for TiO_2−*x*_/Ni-400 (Supplementary Fig. 17), TiO_2−*x*_/Ni-500 (Supplementary Fig. 18), and TiO_2−*x*_/Ni-600 (Supplementary Fig. 19).

In situ Ti *K*-edge XANES were carried out to explore the Ti−O coordination environment. In terms of post-edge feature above 4984 eV, the white line and resolvable peaks of TiO_2−*x*_/Ni-450 show a broad and less pronounced peak than that for the anatase reference, indicating the severely disordered Ti−O coordination environment nature. As for the pre-edge region below 4984 eV, three pre-peaks labeled as *P*_1_, *P*_2_, and *P*_3_ are attributed to the transition of the core electron to the hybridized states of Ti 3*d*4*p*4*s* orbitals^43,48,49^ in Fig. 2f. In the case of TiO_2−*x*_/Ni-450, the *P*_2_ peak at 4970.6 eV increased significantly and distinctly shifted to lower photon energy compared to the standard anatase (4972.2 eV). According to previous works^34^, the peak at 4970.6 eV is ascribed to the five coordinated Ti atoms, which is closely related with the formation of O_*v*_ − Ti^3+^ species. The strong electronic metal-support interaction can occur at the interface accompanied with the electron transfer from O_*v*_ − Ti^3+^ species to the adjacent interfacial Ni atoms to form Ni^*δ−*^ sites. Therefore, abundant Ni^*δ−*^/TiO_2−*x*_ interfacial sites can be efficiently fabricated and stabilized over the TiO_2−*x*_/Ni-450 catalyst. The raising of reduction temperature enhances Ni^*δ−*^ species content, owing to the SMSI between Ni particles and TiO_2−*x*_ support, resulting in the formation of more Ni^*δ−*^/TiO_2−*x*_ interfacial sites.

### The revelation of structure and morphology for various TiO_2−*x*_/Ni Catalysts

**Fig. 3:**
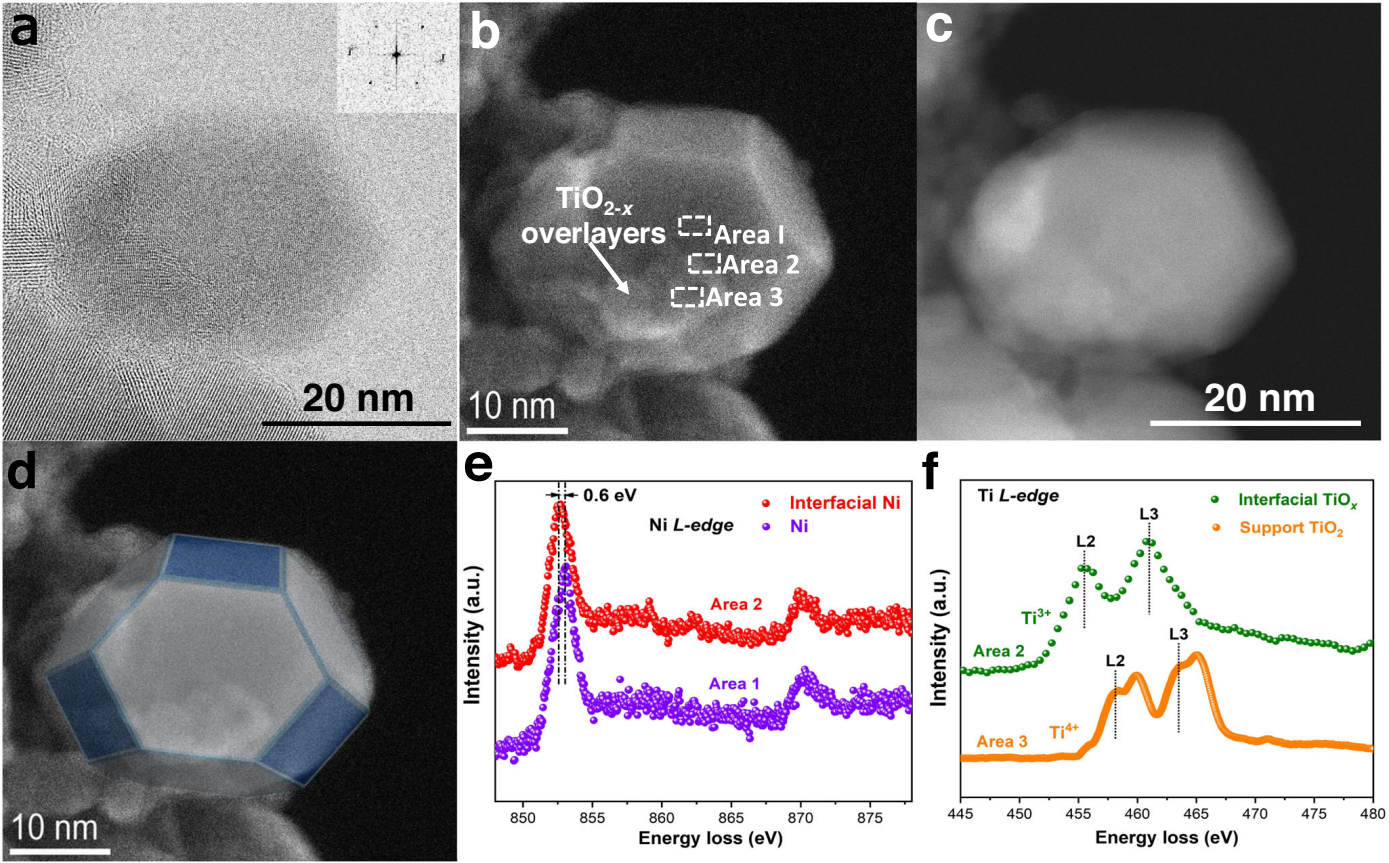
The revelation of structure and morphology for TiO_2−*x*_/Ni-450 catalyst. a STEM-BF (**b**) STEM-SE and (**c**) STEM-ADF images of TiO_2−*x*_/Ni-450 catalyst after reduction in H_2_ atmosphere (10 Pa) at 450 °C. **d** Schematic diagram of Ni particle as shown in **b**. In situ electron energy-loss spectroscopy (EELS) collected at spot I and II. **e** Ni *L*-edge spectra collected at Spot I and II, respectively. **f** Ti *L*-edge spectra collected at Spot I and II, respectively.

In situ environment scanning transmission electron microscope (ESTEM)^50^ was employed to identify the structure and surface topography of the optimal TiO_2−*x*_/Ni-450 and TiO_2−*x*_/Ni-600 catalysts. The detailed schematic diagram of ESTEM can be found in Supplementary Fig. 20. It is generally known that Ni nanoparticles could be oxidized to form superficial NiO layers upon exposure to air at ambient conditions, which inevitably ruined original surface configurations of the reduced catalyst. In order to reveal the intrinsic physicochemical property of Ni-based catalysts, *environmental* electron microscopy is necessary so as to characterize reduced Ni nanoparticles and their surroundings. In a typical process, the passivated TiO_2−*x*_/Ni-450 catalyst was reduced in H_2_ atmosphere (pressure: 10 Pa) at 450 °C for 1 h. The FCC microstructure of the reduced metallic Ni nanoparticle was determined by the inset FFT pattern (Fig. 3a).

In particularly, the advanced ESTEM equipped with a SE (secondary electron) detector enables us to deeply explore surface and morphology information of TiO_2−*x*_/Ni catalyst under the realistic chemical environment. Figure 3b discloses that the reduced Ni particle with a well-ordered shape is partially covered by TiO_2−*x*_ overlayers after the passivated TiO_2−*x*_/Ni-450 reduction at 450 °C. A schematic diagram (Fig. 3d) rationalizes that the surface crystalline plane of the Ni NP is only terminated by low-indexed crystalline planes^51^ and visualizes surface TiO_2−*x*_ deposits on the crystalline surface of the Ni nanoparticles (also seeing Supplementary Fig. 21). In order to evidence the specific interaction between the metal nanoparticle and the oxide, EELS Ni *L*-edge and Ti *L*-edge were also obtained at Area I (Ni particle), Area II (interface), and Area III (TiO_2_ support), so as to distinguish the electronic properties of bulk of Ni NP, interfacial Ni atoms and Ti atoms, and Ti atoms of TiO_2_ support in Fig. 3e, f. It is obvious that the *L*-edge of Ni species at the interface (Area II) show a slight shift (0.6 eV) toward the lower energy compared to the surface Ni species of metal particle (Area I), indicating that the presence of Ni^*δ−*^ species. Abundant Ti^3+^ species^52,53^ can be observed at the interface whilst is absent at the TiO_2_ support (Area III). ESTEM was also carried out over the TiO_2−*x*_/Ni-600 catalyst. After activation in H_2_ atmosphere at 600 °C, the Ni particles were close to be fully covered by several atomic thickness of TiO_2−*x*_ overlayers (Supplementary Fig. 22). STEM-EDS mapping data further confirm the full coverage of TiO_2−*x*_ over the Ni particle (Supplementary Fig. 23). The full coverage significantly hinders the active sites (interfacial Ni^*δ−*^/TiO_2−*x*_ species), resulting in the reduction of catalytic performance (Fig. 1b). It indicates that the higher reduction temperature promotes TiO_2−*x*_ overlayers migrate on the surface of Ni particle *via* SMSI, this is consistent to the surface Ni concentration analysis from CO titration (Supplementary Table 1).

### The investigation of CO hydrogenation process *via* IR spectroscopy

**Fig. 4:**
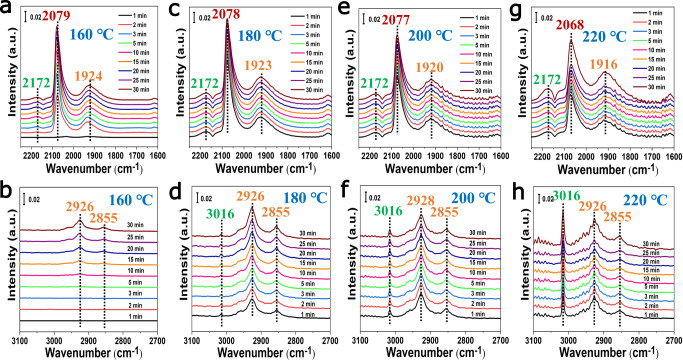
The investigation of CO hydrogenation mechanism *via* in situ IR spectroscopy. In situ time-resolved DRIFTS spectra were carried out over the TiO_2−*x*_/Ni-450 catalyst at **a**, **b** 160 °C, **c**, **d** 180 °C, **e**, **f** 200 °C, **g**, **h** 220 °C, respectively. The corresponding spectra were recorded in 2250 − 1600 cm^−1^ and 3100 − 2700 cm^−1^ at different temperature with the time stream on. From left to right: upon exposure to syngas (CO: H_2_:Ar = 5:10:85; 30 mL/min) at different temperatures as a function of time.

In situ time-resolved DRIFTS were carried out to monitor the dynamic evolution of reactive molecules, including intermediates and products, at different conditions for CO hydrogenation over the TiO_2−*x*_/Ni-450 catalyst (Fig. 4). The primary IR bands at ~2079 cm^−1^ and ~1924 cm^−1^, assigned to linear adsorbed CO species on Ni defect sites and bridge adsorbed species on Ni^*δ−*^ sites, respectively, are observed at 160 °C under the reaction condition in Fig. 4a, which is in consistent with the result in CO atmosphere in Supplementary Fig. 24. The IR bands at ~2926 cm^−1^ and ~2855 cm^−1^, which are ascribed to the asymmetric *v*_as_(C − H) and symmetric *v*_s_(C − H) stretching vibration of CH_2_* species^54^, increase gradually with the extension of exposure time (Fig. 4b), indicating the formation of C_2+_ paraffin products. However, no obvious IR band at ~3016 cm^−1^ (assigned to the *v*_(C−H)_ stretching vibration^32,55^ of CH_4_ molecules) can be found for the TiO_2−*x*_/Ni-450 catalyzing CO hydrogenation. This demonstrates that C_2+_ hydrocarbon products are the primary product rather than CH_4_ for CO hydrogenation at 160 °C. When the reaction temperature raises up to 180 °C, two types of IR bands at ~2079 cm^−1^ and ~1924 cm^−1^ show inconspicuous change (Fig. 4c). A very weak IR band at ~3016 cm^−1^ was observed assigned to the C − H stretching mode of CH_4_ molecules. The IR bands at ~2926 cm^−1^ and ~2855 cm^−1^ display obvious increment as shown in Fig. 4d, indicating the enhancement of catalytic performance of FTS with the formation of much more C_2+_ paraffin product. The similar phenomenon could also be found at 200 °C in Fig. 4e, f. With the reaction temperature sequentially increasing to 220 °C, the IR bands at ~2068 cm^−1^ and ~1916 cm^−1^ in Fig. 4g decreased remarkably due to the substantial increment of catalytic performance. The C − H stretching mode of CH_4_ molecules at ~3016 cm^−1^ increase substantially accompanied with the presence of abundant CH_2_* species at ~2926 cm^−1^ and ~2855 cm^−1^ in Fig. 4h, indicating the simultaneous enhancement of catalytic activity for the methanation and C_2+_ paraffin products *via* FTS, which is in good agreement with the experiment results (Supplementary Table 3).

### Reaction mechanisms identification by DFT calculations

**Fig. 5:**
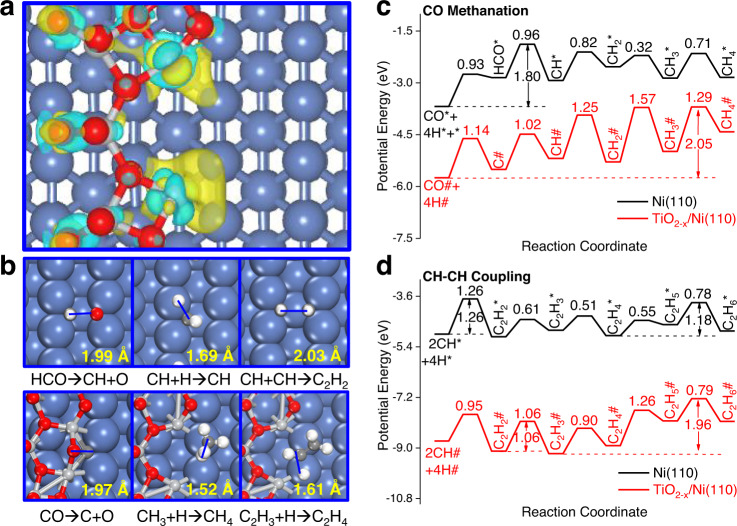
DFT optimized structures and reaction mechanism studies. **a** Differential charge densities of Ti_6_O_11_/Ni(110) surface. The light blue and yellow contours (isosurface = 0.006 e/Å^3^) represent the charge depletion and charge accumulation, respectively. **b** Key transition states configurations for CO hydrogenation towards CH_4_ and C_2_H_6_ products over Ni(110) (Top panel) and Ti_6_O_11_/Ni(110) (Bottom panel) surfaces. The solid blue line is drawn to signify the bond dissociation or formation. The bond distances at the transition states for CO hydrogenation are indicated in Å. The blue, light gray, gray, red and white spheres are Ni, Ti, C, O and H atoms, respectively. **c**, **d** Potential energy diagrams for CO methanation and C_2_H_6_ formation *via* CH-CH coupling and hydrogenation over Ni(110) and Ti_6_O_11_/Ni(110) surfaces, respectively. The elementary reaction barriers are indicated in eV and all the energies in (**c**) and (**d**) are calculated with respect to CO + 2H_2_ and 2CO + 3H_2_ in the gas phase, respectively.

Density functional theory (DFT) calculations were performed to understand CO hydrogenation mechanism over TiO_2−*x*_/Ni(110) catalyst. Here, Ti_6_O_11_/Ni(110) surface, where one layer Ti_6_O_11_ cluster kept the features of TiO_2_(101) surface was deposited on Ni(110) surface, were used to model interfacial TiO_2−*x*_/Ni(110) catalysts (Supplementary Fig. 25). The open Ni(110) surface was selected here because it exposes the Ni particles and contains many under-coordinated surface Ni atoms exhibiting high activity for CO activation^20^. All Ti atoms and eight O atoms in Ti_6_O_11_ cluster tend to bind Ni(110) surface resulting in a great charge transfer between Ti_6_O_11_ cluster and Ni(110) surface (Fig. 5a). Two Ti^3+^ cations always generate accompanied with the formation of one oxygen vacancy (O_*v*_) at the interface of Ti_6_O_11_/Ni(110) surface. Bader charge analysis indicates that the interficial Ni atoms at V_O_ nearby have negative charge states (Supplementary Fig. 25 and Supplementary Table 7), which is corroborated by our experimental characterizations. The formation of interfacial Ni^*δ−*^/TiO_2−*x*_ site over Ti_6_O_11_/Ni(110) surface could show different catalytic performance of CO hydrogenation as compared with Ni(110) surface attributed from their different geometric and electronic structures discussed below.

CO hydrogenation is initialized by CO activation and hydrogenation in the generation of CH_*x*_ (*x* = 0–3) monomers, which can be further hydrogenated to methane (CH_4_) and/or coupled with each other forming C_2+_ alkanes/olefines (Fig. 5b). Ti_6_O_11_/Ni(110) and Ni(110) surfaces exhibit different CO activation and further hydrogenation mechanisms (Supplementary Fig. 26). The overall activation barrier for hydrogen-assisted CO dissociation has the highest activation barrier (*E*_a_ = 1.80 eV) and can be regarded as the rate-determining step (RDS) for CH_4_ and C_2_H_6_ formation over Ni(110) surface. The elementary reaction steps in CH hydrogenation towards CH_4_ are neutral with the highest activation barrier of 0.82 eV on Ni (110) surface. However, C_2_H_6_ formation *via* CH-CH, CH-CH_2_ and CH_2_-CH_2_ coupling have comparable overall higher activation barriers of 1.21–1.26 eV over Ni (110) surface (Fig. 5c, d and Supplementary Fig. 26). Therefore, CH_4_ rather than C_2_H_6_ is often the dominant product for CO hydrogenation on Ni-based surface in line with our experimental observations.

Different from Ni(110) surface, direct CO dissociation path (*E*_a_ = 1.14 eV) is more feasible than hydrogen-assisted CO activation *via* formyl (HCO) route monomer by 0.43 eV over Ti_6_O_11_/Ni(110) surface. Therefore, Ti_6_O_11_/Ni(110) surface has a much higher activity than Ni(110) surface by 0.55/0.54 eV for CO activation *via* COH/HCO intermediates more favorable for Ni catalyst (Supplementary Fig. 26). The higher activity of Ti_6_O_11_/Ni(110) for CO activation can be attributed to the strong binding strength between Ti atom and O atom lowering the corresponding transition state energy and reaction energy of direct CO dissociation.

The hydrogenation of strongly adsorbed dissociated C atoms towards CH_4_ at the interfacial Ni^*δ−*^/TiO_2−*x*_ site of Ti_6_O_11_/Ni(110) is highly endothermic thus resulting in a low activity for CH_4_ formation with high activation barrier of 2.05 eV (Fig. 5c). In contrast, C_2_H_4_ formation *via* CH-CH coupling step is much more favorable in the generation of C_2_H_4_ intermediate than CH_4_ product at the interfacial Ni^*δ−*^/TiO_2−*x*_ site of Ti_6_O_11_/Ni(110) surface with an activation barrier of 1.06 eV (Fig. 5d and Supplementary Fig. 27). The hydrogenation of formed C_2_H_4_ intermediate in the generation of C_2_H_6_ has to overcome a high activation barrier of 1.67 eV at the the interfacial Ni^*δ−*^/TiO_2−*x*_ site of Ti_6_O_11_/Ni(110) surface (Supplementary Fig. 27) that C_2_H_4_ intends to migrate to Ni (110) surface for further hydrogenation towards C_2_H_6_ with a lower overall activation barrier of 1.18 eV. In other words, Ni^*δ−*^/TiO_2−*x*_ site of Ti_6_O_11_/Ni(110) can be used for CO activation and CH-CH coupling in the formation of C_2_H_4_, whereas Ni(110) surface serves as the hydrogenation platform in the formation of C_2_H_6_ product. Both CO activation and C_2_H_6_ formation control CO hydrogenation activity at the interfacial Ni^*δ−*^/TiO_2−*x*_ site of Ti_6_O_11_/Ni(110) surface with the activation barriers lower than those on Ni(110) surface by 0.62 eV at least. Ultimately, Ni^*δ−*^/TiO_2−*x*_ site of Ti_6_O_11_/Ni(110) surface shows higher activity and selectivity of CO hydrogenation towards C_2_H_6_ as compared with Ni(110) surface with CH_4_ as the main product. Our theoretical calculations are corroborated by experimental measurements that the interfacial site at TiO_2−*x*_/Ni-450 catalyst plays an important role in CO hydrogenation towards ethane. TiO_2−*x*_/Ni-450 is more active than 15% Ni/SiO_2_ for CO hydrogenation towards ethane and TiO_2−*x*_/Ni-450 catalyst has a higher TOF and lower apparent activation barrier (Supplementary Table 1 and Supplementary Fig. 11).

## Discussion

In summary, an interfacial TiO_2−*x*_/Ni catalyst was fabricated derived from LDHs precursor displaying a high CO conversion of ~19.8% and ~64.6% selectivity for C_2+_ paraffin at atmosphere pressure for FTS process. Advanced electron microscopy and in situ characterization were executed to verify that disordered titania overlayers modulated the surface of Ni particles to generate abundant Ni^*δ−*^/TiO_2−*x*_ interfacial sites by SMSI. DFT calculations reveal that TiO_2−*x*_/Ni catalyst exhibits extraordinary high activity and selectivity for CO hydrogenation towards carbon coupling products originated from catalytic synergistic effect, namely, the interfacial Ni^*δ−*^/TiO_2−*x*_ site enhances CO dissociation and facilitates the growth of C-C chain. The present work not only discovers a new-type SMSI catalyst with the unique interfacial structure for syngas conversion at atmosphere pressure, but also insight into the interfacial synergistic catalysis driven by SMSI effect.

## Methods

### Catalyst preparation

Firstly, the ultrathin NiTi-LDHs precursor was prepared *via* the revised hydro-thermal synthesis approach according to our previous work^34^. In a typical process, Ni(NO_3_)_2_·6H_2_O (0.006 mol), HCl (0.25 mL), TiCl_4_ (0.25 mL), and urea (0.1 mol) were dissolved into deionised water (100 mL) under vigorous stirring for 24 h at 95 °C; The resulting solid was centrifuged washed thoroughly and dried at 60 °C overnight; Afterward, NiTi-LDHs nanoplates were calcinated under air with the heating rate of 2 °C/min in the muffle furnace to obtain the mixed oxide NiTi-MMO (the mixture of NiO and TiO_2_). Subsequently, the as-obtained NiTi-MMO mixed oxides were reduced under 10%H_2_/Ar with a flow rate of 20 mL/min and a heating rate of 2 °C/min for 4 h at different temperature, such as 300, 350, 400, 450, 500, 550, and 600 °C, which were denoted as TiO_2−*x*_/Ni-300, TiO_2−*x*_/Ni-350, TiO_2−*x*_/Ni-400, TiO_2−*x*_/Ni-450, TiO_2−*x*_/Ni-500, TiO_2−*x*_/Ni-550, and TiO_2−*x*_/Ni-600, respectively. For comparison, the 15% Ni-SiO_2_ catalyst was fabricated by the traditional impregnation approach. In a typical process, an aqueous solution containing of 0.55 mmol Ni(NO_3_)_2_·6H_2_O was mixed with the SiO_2_ support (1 g), the suspension liquid kept stirring for 3 h, and then was transformed into the drying oven at 120 °C overnight. The as-synthesized NiO-SiO_2_ was reduced under 10% H_2_/Ar with a flow rate of 20 mL/min for 4 h at 450 °C (a heating rate: 5 °C/min), so as to obtain the 15% Ni/SiO_2_ catalyst. The loading of Ni was determined *via* inductively coupled plasma atomic emission spectrometry (ICP).

### Catalyst characterization

Powder XRD experiments were carried out on a Rigaku XRD-6000 diffractometer with Cu *K*α radiation (Condition: *λ* = 0.15418 nm, 40 kV, and 30 mA; Scan rate: 5° min^−1^) and a 2*θ* angle ranging from 3° to 90°. The crystal structures of components could be identified based on JCPDS standard cards. Scanning electron microscope (SEM; Zeiss SUPRA 55) was carried out with an accelerating voltage of 20 kV. High-resolution transmission electron microscopy (HRTEM) was performed on a JEM-3010 at an accelerating voltage of 200 kV. X-ray photoelectron spectroscopy (XPS) measurements were conducted on an Axis Ultra Imaging Photoelectron Spectrometer equipped with Al *K*α (1486.7 eV) quartz monochrometer source. The freshly prepared catalysts were transferred to the measurement chamber without exposure to air. The binding energy was corrected by setting active carbon sp2 C 1 s of 284.5 eV as the reference. The XAFS spectra of Ni *K-*edge (8333 eV) and Ti *K*-edge was collected at 1W1B beamline of Beijing Synchrotron Radiation Facility (BSRF), Institute of High Energy Physics (IHEP), Chinese Academy of Sciences (CAS). The beam was tuned by the Si (111) double-crystal monochromators. The energies were calibrated according to the absorption edge of pure Ni foil and Ti foil. The typical energy of the storage ring was 2.5 GeV with a maximum current of 250 mA. The powdered sample was first pressed into the sheet and loaded into a reactor cell equipped with polyimide windows. The sample sheet was reduced in a H_2_/He stream at different temperatures ranging from 400 to 600 °C in the reactor cell, followed by flushing (50 mL min^−1^) with high purity He for 1.0 h, and then the EXAFS spectra at the Ni *K*-edge and Ti *K*-edge were collected respectively. In situ diffuse reflectance infrared Fourier-transform spectroscopy (DRIFTS) were carried out in a modified in situ reaction cell on a VERTEX 70 spectrometer equipped with a MCT narrow-band detector, with a resolution of 4 cm^−1^.

### CO DRIFTS experiment

In situ DRIFTS spectra were performed to reveal the CO chemisorption state at different Ni sites over various TiO_2−*x*_/Ni catalysts (Fig. 2c). As for CO-DRIFTS on TiO_2−*x*_/Ni-450 catalyst, the powdered sample (NiTi-MMO) was filled into the reactor, and was pre-reduced in 10%H_2_ (Ar balance) at 450 °C for 4 h and flushed with high purity Ar for 30 min. Then, 5% CO (Ar balance) was introduced at 25 °C, respectively, for 30 min. Afterward, CO-DRIFTS spectra were collected after flushing with Ar for another 30 min. The CO-DRIFTS experiments for TiO_2−*x*_/Ni-300, TiO_2−*x*_/Ni-350, TiO_2−*x*_/Ni-400, TiO_2−*x*_/Ni-500, TiO_2−*x*_/Ni-550, and TiO_2−*x*_/Ni-600 samples were carried out *via* the similar experimental processes.

### In situ time-resolved DRIFTS experiments

(a) In situ time-resolved DRIFTS were also carried out to further monitor the dynamic evolution of reactive molecules, active intermediates, and products at different conditions for CO hydrogenation over the fresh TiO_2−*x*_/Ni-450 catalyst (Fig. 4). As for TiO_2−*x*_/Ni-450 catalyst, the powdered sample (NiTi-MMO) was filled into the reactor, and was pre-reduced in 10%H_2_ (Ar balance) at 450 °C for 4 h and flushed with high purity Ar for 30 min. Afterwards, the temperature was decreased to 160 °C. Then, syngas (CO:H_2_:Ar = 5:10:85; 30 mL/min) was introduced into this system at the temperature for 30 min. Simultaneously, in situ time-resolved DRIFTS spectra were collected with the time stream on. In addition, the temperature was increased to 180 °C, 200 °C, and 220 °C, respectively. The corresponding time-resolved DRIFTS spectra were collected at different temperatures for 30 min as a function of the time. (**b**) In situ time-resolved CO-DRIFTS spectra were also carried out at 160 °C, 180 °C, 200 °C, and 220 °C, respectively, according to the above similar experiment processes.

### ESTEM experiments

The sample was directly dispersed in a mems chip used for an in-situ environmental STEM experiment (not dispersion in ethanol). After inserting a heating holder equipped with the prepared chip inside the TEM, H_2_ gas was introduced into the TEM column and the pressure was maintained at 2 Pa. The temperature was gradually increased from 200 to 450 °C. The structural evolution was recorded under the STEM mode. All EELS spectra were collected during the in-situ experiment (in presence of H_2_). Reference spectra (TiO_2_ and metallic Ni) were recorded at 450 °C under H_2_ atmosphere. EELS spectrum imaging (SI) under STEM mode was performed in the targeted area to examine the ELNES fine structure of Ni and titania. Instrumental information: Hitachi HF5000 probe-corrected environmental S/TEM, cold FEG, accelerating voltage 200 kV. The instrument enables simultaneously imaging ABF, ADF and SE images of an identical particle. EELs collected Gatan 965 energy filter.

### CO chemisorption

This experiment was performed in a quartz tube reactor on an Automated Catalyst Characterization System (AutoChem 2920) from MICROMERITICS equipped with an on-line mass spectrometry (MS). In a typical process, 100 mg of sample was firstly pre-reduced at 300 °C, 350 °C, 400 °C, 450 °C, 500 °C, 550 °C, and 600 °C for 4 h, respectively, followed by flushing with high purity He for 1.0 h and then the temperature was decreased to 200 K. Subsequently, successive pulses of CO were introduced, using He as the carrier gas (50 mL min^–1^), until a stable mass signal of CO was obtained. The dispersion of metal Ni (*D*%, Eq. 1) was calculated on the basis of CO chemisorption value^34^:

1 $$D\left(\%\right)=\frac{{SF}\times {V}_{{{{{{\rm{ad}}}}}}}\times {M}_{{{{{{\rm{Ni}}}}}}}}{{m}_{{{{{{\rm{s}}}}}}}\times {W}_{{{{{{\rm{s}}}}}}}\times {V}_{{{{{{\rm{m}}}}}}}\times {d}_{{{{{{\rm{r}}}}}}}}\times 100$$

Where *M*_*Ni*_, *m*_*s*_, *V*_*ad*_, and *W*_*s*_ are the molecular weight of Ni, the weight of sample (g), the volume (mL) of CO chemisorption, and the weight fraction of Ni, respectively. *V*_*m*_ is the molar volume of CO (22414 mL mol^−1^) at the standard temperature and pressure (STP); *dr* is the reduction degree of Ni; *SF* is the stoichiometric factor, which is assumed as 1:1 for CO:Ni.

### Catalyst performance measurements

The reactions were carried out in a multichannel fixed-bed reactor with a 10 mm inner diameter quartz tube inside. 120 mg catalyst sieved into 40–60 mesh was diluted with quartz sand. Prior to catalytic test, the catalyst was activated with 10% H_2_/N_2_ (20 mL min^−1^) at 300, 350, 400, 450, 500, 550, 600 ^o^C for 4 h. The reaction was conducted in bar syngas (32%CO, 64%H_2_, 4%Ar) at a flow rate of 20 mL min^−1^ at 200 and 220 ^o^C, respectively. The FTS products were analyzed by Agilent 7890 A gas chromatography. Permanent gas and CO_2_ were analyzed by 5 A molecular sieve column and Plot Q column with thermal conductivity detector and low hydrocarbons were analyzed by Al_2_O_3_ capillary column with hydrogen flame ionization detector.

The CO conversion, productive selectivity, reaction rate, and catalytic activity were calculated based on the following formula:

2 $${\mbox{CO conversion was defined as}}:{\mbox{CO conversion}}\,(\%)=\frac{{F}_{{CO},{in}}-{F}_{{CO},{out}}}{{F}_{{CO},{in}}}\times 100$$

3 $${\mbox{Product selectivity was defined as}}:{\mbox{Selectivity}}\,({\mbox{mol}}\%)=\frac{{F}_{{Ci}}\times i}{{F}_{{CO},{in}}-{F}_{{CO},{out}}}\times 100$$

4 $${\mbox{Reaction Rate}}=\frac{{GHSV}\times {CO} \, {Conversion}\,\times \,{CO}\,{Concentration}}{22400\,\times \,{\omega }_{{Ni}}}$$

5 $${\mbox{C}}{\mbox{a}}{\mbox{talytic activity}}=\frac{{Reaction}\,{Rate}\,\times \,{M}_{{Ni}}}{3600\,\times \,{{Ni}}_{{dispersion}}}$$

Where *F* is the moles of CO and product *Ci* (CO_2_ and hydrocarbon) containing *i* carbon atoms; *GHSV* is the hourly space velocity (10000 mL g_cat_^−1^ h^−1^), and *ω*_*Ni*_ is the mass fraction of Ni, which was detected by ICP-OES; *M*_*Ni*_ is the atomic mass of Ni (58.69 g·mol^−1^); *Ni*_*dispersion*_ is the dispersion of metal Ni for different Ni-based catalysts calculated from the Eq.1 above.

## Supplementary information

Supplementary Information

## Data Availability

The data that support the plots within this paper and other finding of this study are available from the corresponding author upon request. Source data are provided with this paper.

## Source data

Source Data
