# Supplementary material for: Boosting CO hydrogenation towards C2+ hydrocarbons over interfacial TiO2−x/Ni catalysts
Source: Nat Commun. 2022 Nov 7;13:6720. doi: 10.1038/s41467-022-34463-7 (PMC9640681; doi:10.1038/s41467-022-34463-7)
Supplement: Supplementary file 1 — Supplementary Information [file 41467_2022_34463_MOESM1_ESM.pdf]

Supporting Information

Boosting CO hydrogenation towards C_{2+} hydrocarbons over interfacial TiO_{2-x}/Ni catalysts

Ming Xu,[#1,2] Xuetao Qin,[#1] Yao Xu,[1] Xiaochen Zhang,[1] Lirong Zheng,[3] Jin-Xun Liu,[*4] Meng Wang,[*1] Xi Liu,[*5, 6] Ding Ma[*1]

[1]Beijing National Laboratory for Molecular Engineering, College of Chemistry and Molecular Engineering, Peking University, Beijing 100871, P. R. China

[2]State Key Laboratory of Chemical Resource Engineering, Beijing University of Chemical Technology, Beijing 100029, P. R. China

[3]Institute of High Energy Physics, the Chinese Academy of Sciences, Beijing 100049, P. R. China

[4]Department of Chemical Physics, University of Science and Technology of China. Hefei 230026, P. R. China

[5]School of Chemistry and Chemical Engineering, In-situ Center for Physical Sciences, Shanghai Jiaotong University, Shanghai 200240, P. R. China

[6]Syncat@Beijing, Synfuels China Co., Ltd, Beijing 101400, P. R. China

Ming Xu and Xuetao Qin contributed equally to this work.

Correspondence and requests for materials should be addressed to D. M. (Email: dma@pku.edu.cn; liuxi@sjtu.edu.cn; m.wang@pku.edu.cn; jxliu86@ustc.edu.cn)

This file includes:

Supplementary Methods

Supplementary Discussion

Supplementary Figures 29

Supplementary Tables 7

Supplementary References

1. Supplementary Methods

Calculations methods. All first-principles density function theory (DFT) calculations were performed by using projector augmented wave (PAW)[1] implemented in the Vienna ab initio simulation package (VASP).[2,3] We adopted the Perdew-Becke-Ernzerhof (PBE)[4] Generalized Gradient Approximation (GGA) for the exchange-correlation functional. The total energy convergence threshold was set to 10^{-4} eV, and the geometries were considered to be fully relaxed when the forces for each atom were less than 0.05 eV/Å. The cutoff energy was set at 400 eV. The Monkhorst-Pack[5] k-points sampling of ~ 0.05 $Å^{-1}$ were used bulk and surface calculations. The strongly correlated 3*d* electron of Ti was treated with the GGA + *U* correction with U_{eff} = 4.2 eV which is suggested in other theoretical works[6,7]. The determined equilibrium lattice constants for Ni and TiO_2 are a=b=c= 3.52 Å and a=b=3.882 Å with c= 9.639 Å, respectively.

A seven-layer slab within a $p(2 \times 2)$ surface unit cells was used to model Ni (110) surface. The bottom four Ni layers are fixed at their bulk positions and the topmost three layer and the adsorbates were allowed to fully relax. One layer Ti_6O_{11} cluster kept the features of TiO_2(101) surface deposited on $p(5\times5)$ unit cells of Ni(110) surface was adopted to model TiO_{2-x}/Ni(110) catalyst. The Monkhorst-Pack[4] k-points sampling of 4×6×1 and Γ point were used for the calculations of Ni(110) and TiO_{2-x}/Ni(110) surfaces, respectively. The topmost two Ni layers and Ti_6O_{11} cluster were allowed to fully relax. The improved force reversed method[8] was used to locate the transition states (TS). Some of the TSs are verified by climbing-image nudged elastic band (CI-NEB) methods[9,10]. The zero-point energy correction was not taken into account in the present work. The activation barriers are calculated as the energies difference between the transition state and the initial/final state. The separate most stable adsorbed fragments on the surface are chosen as the initial and final states.

2. Supplementary Figures

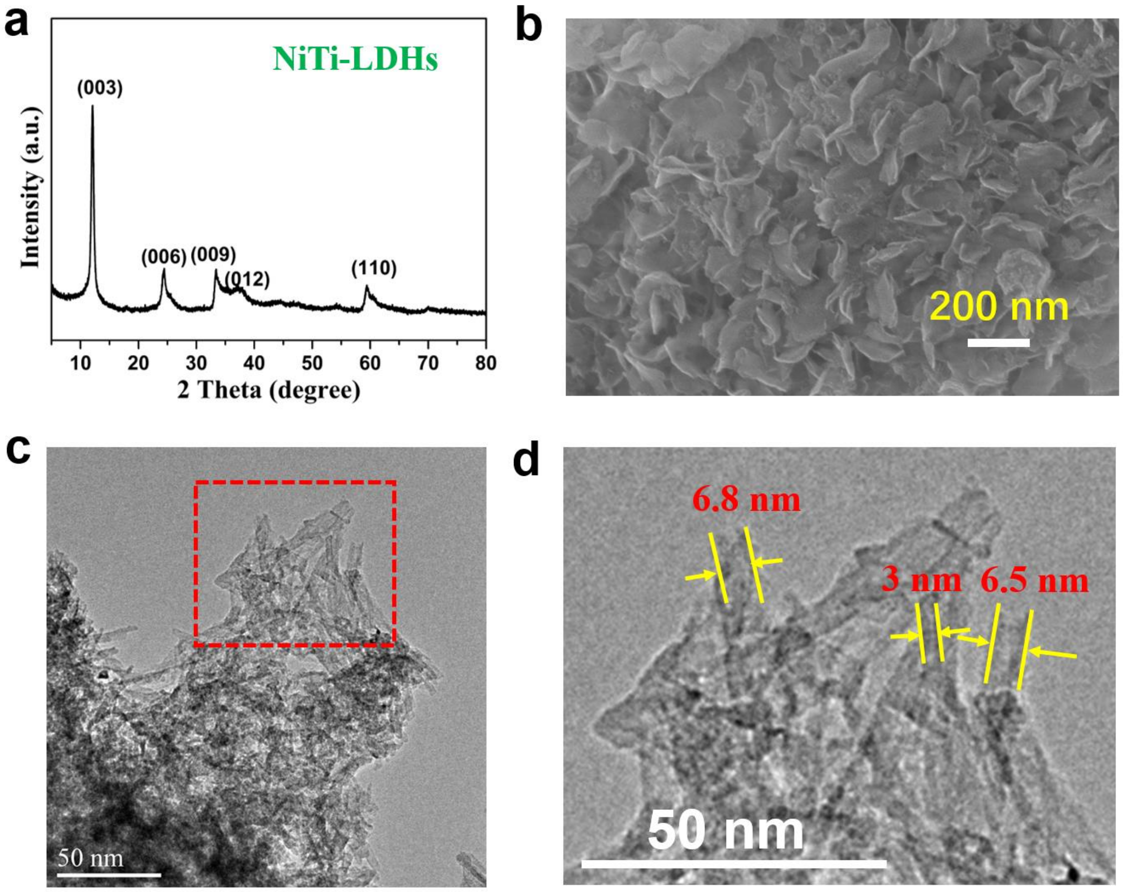

Supplementary Figure 1 | Structure and morphology characterization of NiTi-LDHs. a, XRD patterns, **b,** SEM images, **c,** HRTEM images of NiTi-LDHs, and **d,** The enlarged image of the box area in **c**.

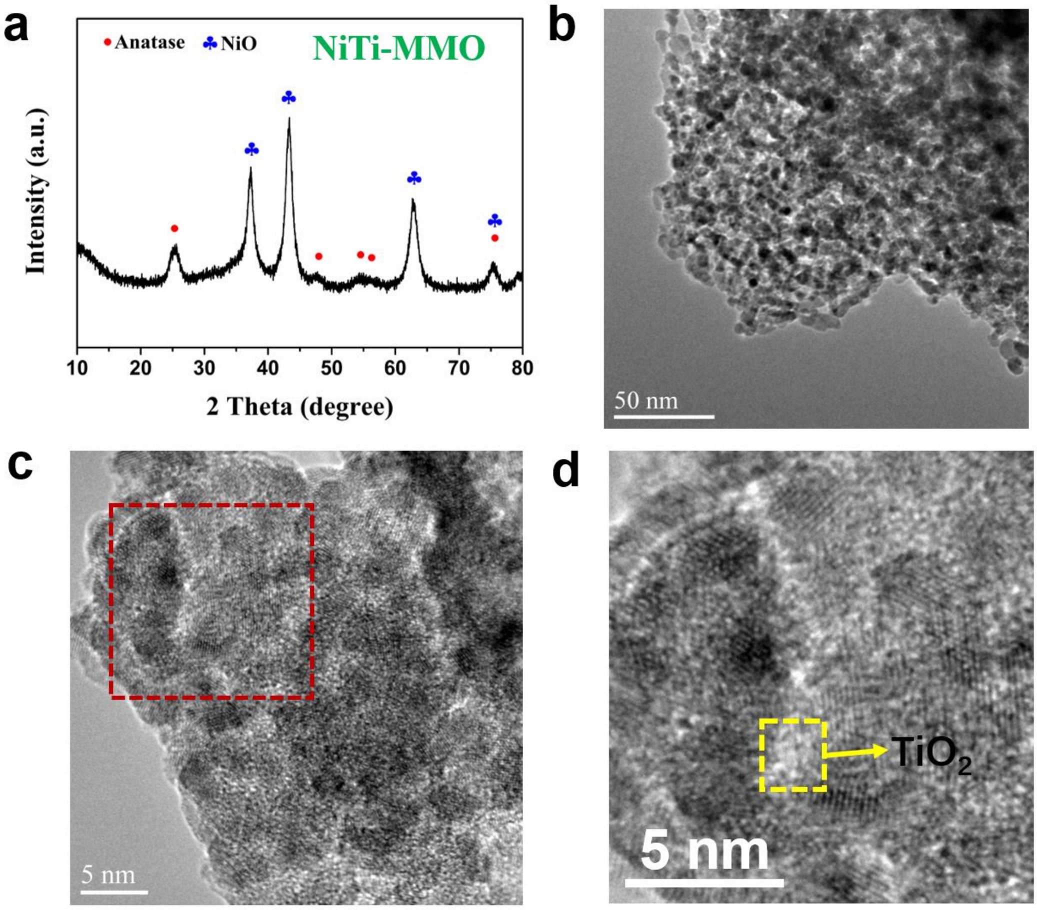

Supplementary Figure 2 | Structure and morphology characterization of NiTi-MMO. a, XRD patterns, **b,** TEM images, **c,** HRTEM images of NiTi-MMO, and **d,** The enlarged image of the box area in **c**.

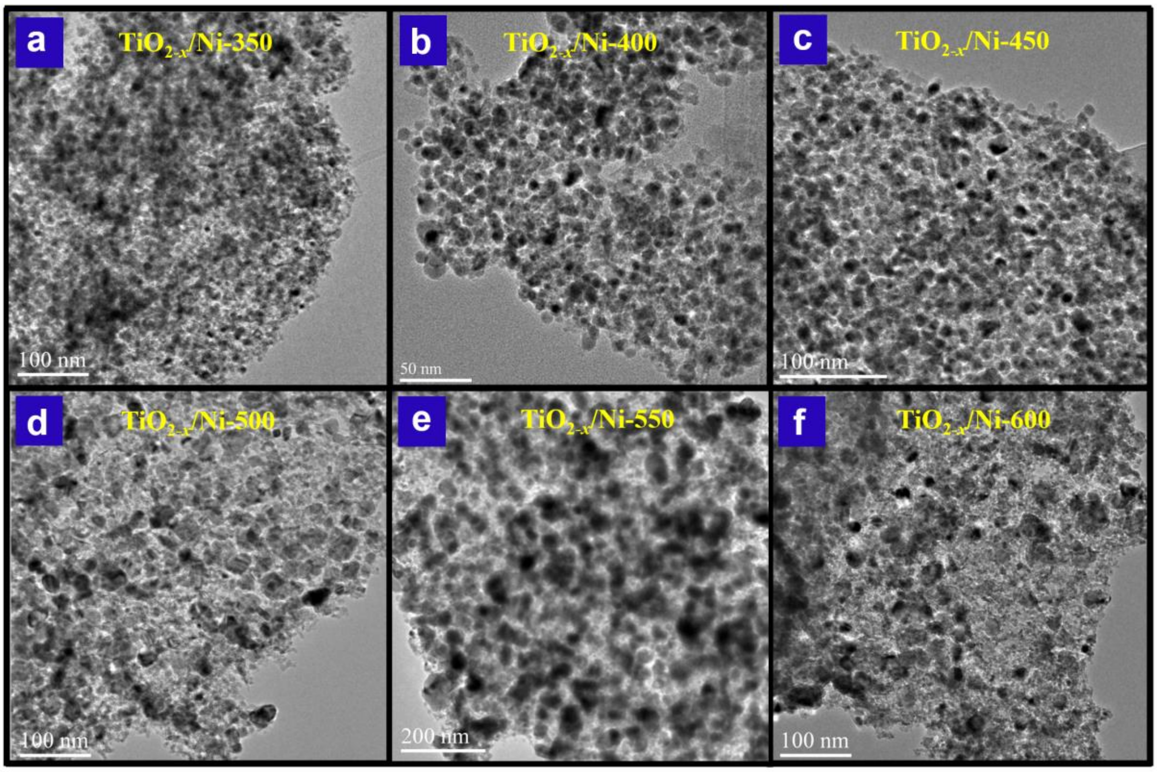

Supplementary Figure 3 | HRTEM images of various TiO_{2-x}/Ni catalysts. HRTEM images of **a,** $NiTiO_{2-x}$-350, **b,** TiO_{2-x}/Ni-400, **c,** TiO_{2-x}/Ni-450, **d,** TiO_{2-x}/Ni-500, **e,** TiO_{2-x}/Ni-550, and **f,** TiO_{2-x}/Ni-600 catalysts.

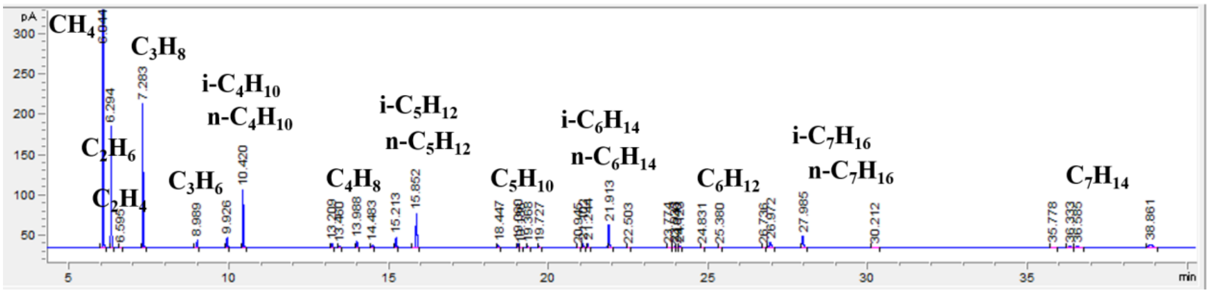

Supplementary Figure 4 | Products distribution of CO hydrogenation. Chromatogram of products generated from the CO hydrogenation over the $TiO_{2\text{-}x}$/Ni-450 catalyst under atmosphere pressure at 220 °C.

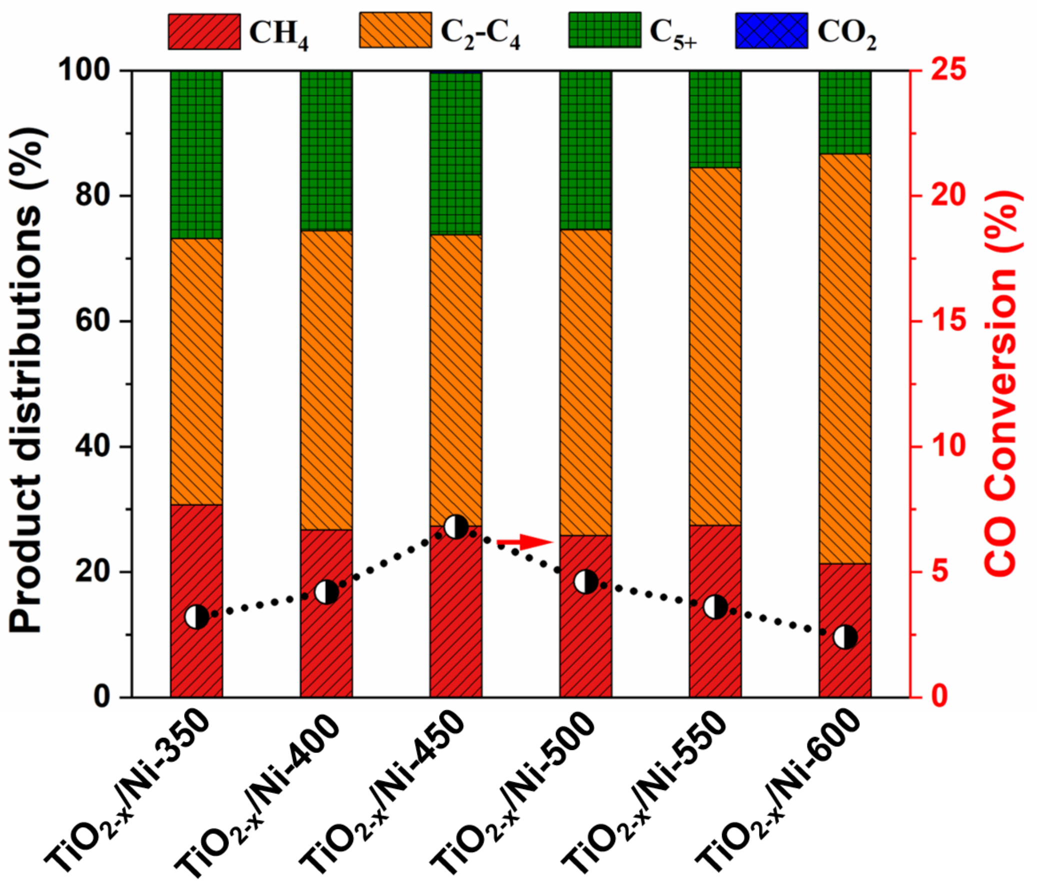

Supplementary Figure 5 | Catalytic performance of CO hydrogenation over various TiO_{2-x}/Ni catalysts. The conversion and product distributions over various TiO_{2-x}/Ni catalysts at 200 ℃. Reaction conditions: catalysts (120 mg), 1 bar, syngas (CO:H_2:Ar=32:64:4; 20 mL/min).

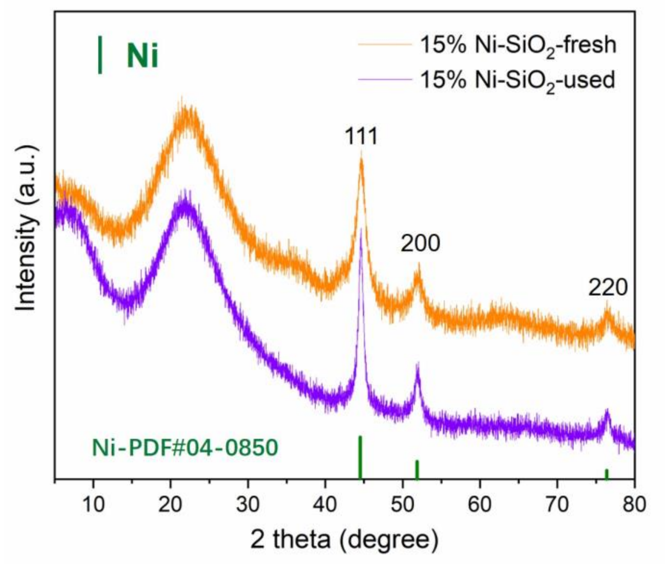

Supplementary Figure 6 | XRD patterns of 15% Ni/SiO_2 before and after reaction. XRD patterns of 15% Ni/SiO_2 after reduction at 450 ℃ and after reaction at 220 ℃ for 12h.

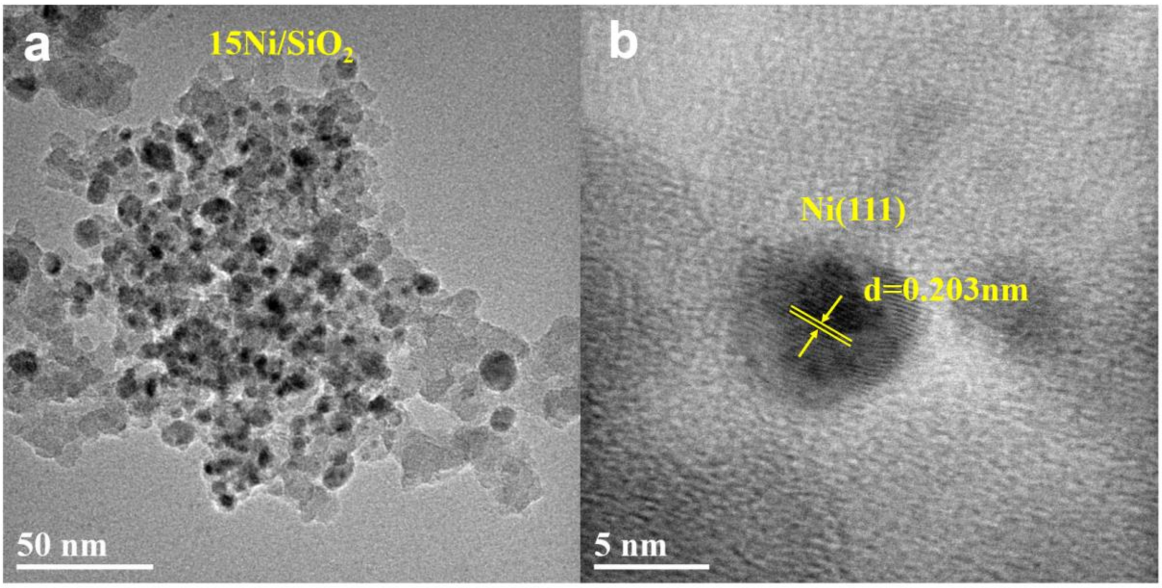

Supplementary Figure 7 | TEM and HRTEM images of 15% Ni/SiO$_2$. a, TEM image and **b,** HRTEM image of 15% Ni/SiO$_2$ catalyst after reduction in hydrogen at 450 °C for 4h.

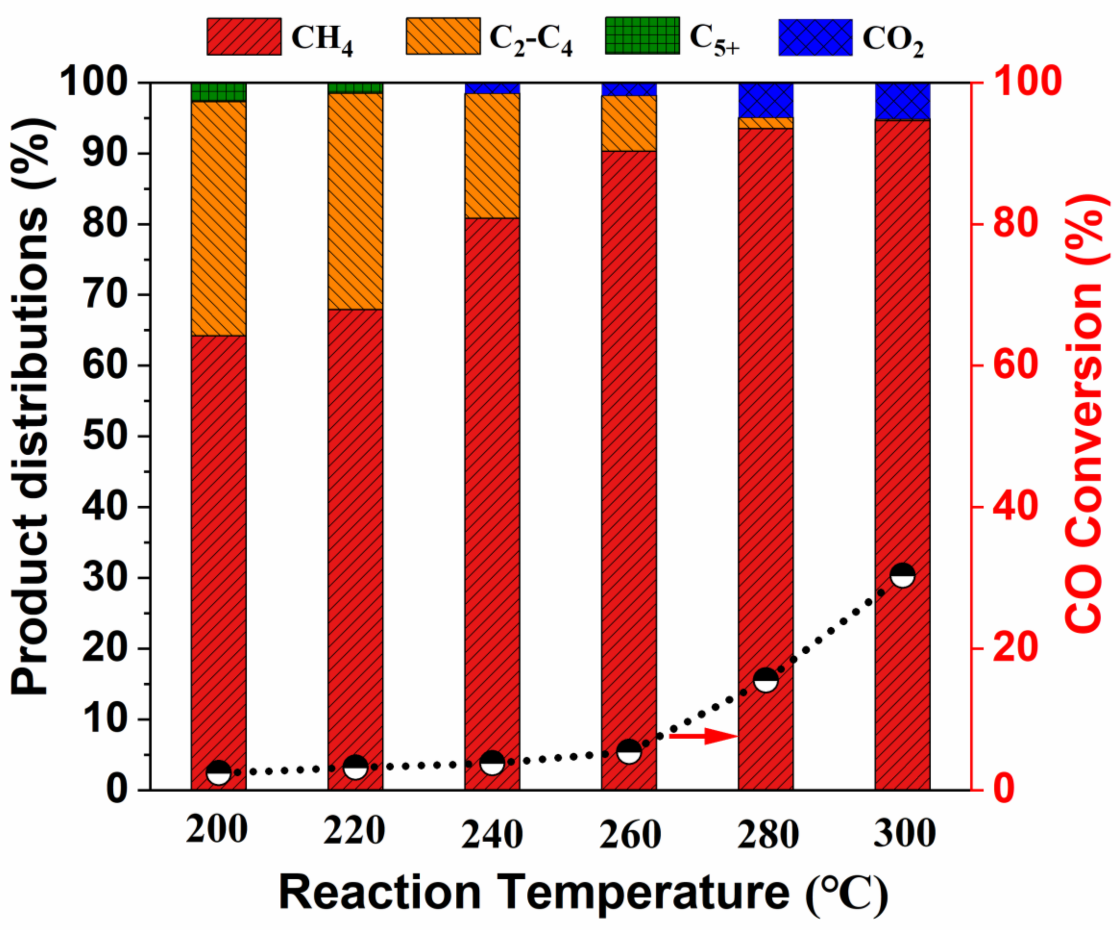

Supplementary Figure 8 | Catalytic performance of CO hydrogenation over 15% Ni/SiO_2 at different temperature. The conversion and product distribution on 15% Ni/SiO_2 at 200, 220, 240, 260, 280, and 300 °C, respectively. Reaction conditions: catalysts (120 mg), 1 bar, syngas (CO:H_2:Ar=32:64:4; 20 mL/min).

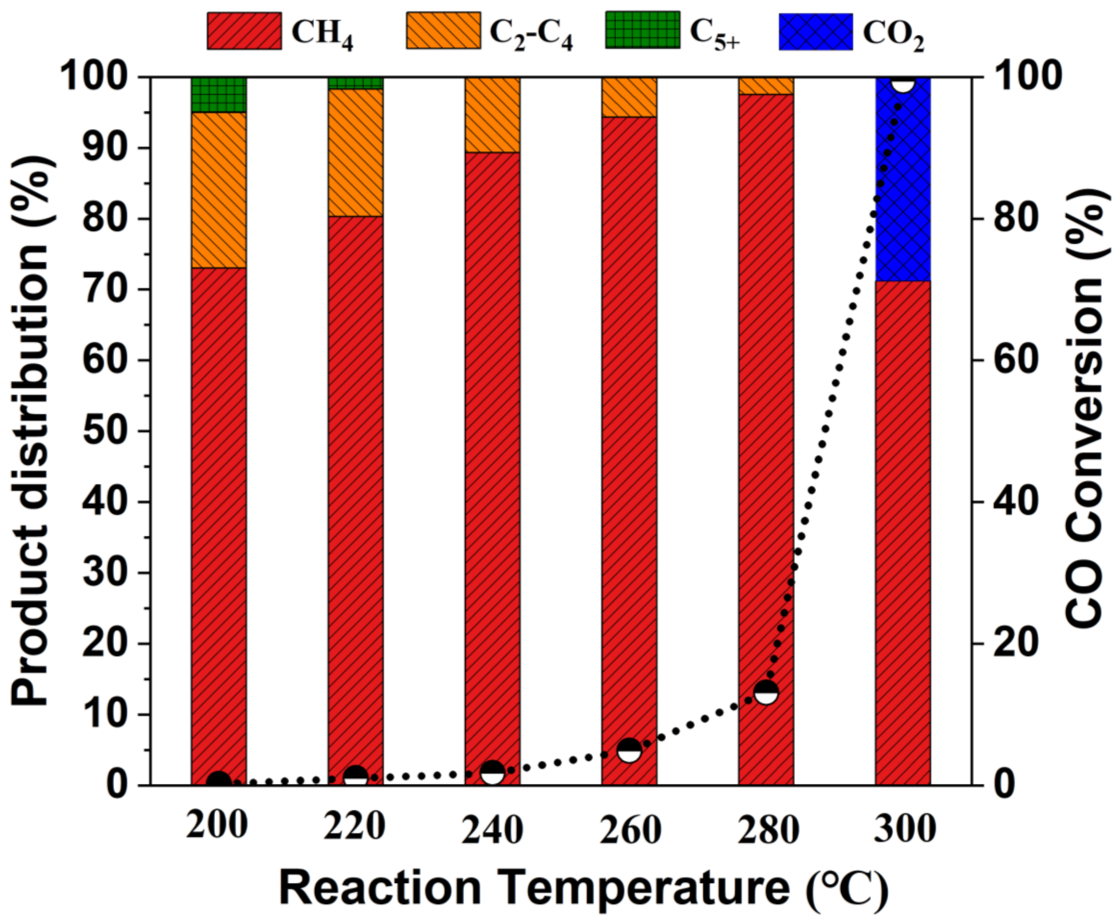

Supplementary Figure 9 | Catalytic performance of CO hydrogenation over 30% Ni/SiO_2 at different temperature. The conversion and product distribution on 30% Ni/SiO_2 at 200, 220, 240, 260, 280, and 300 °C, respectively. Reaction conditions: catalysts (120 mg), 1 bar, syngas (CO:H_2:Ar=32:64:4; 20 mL/min).

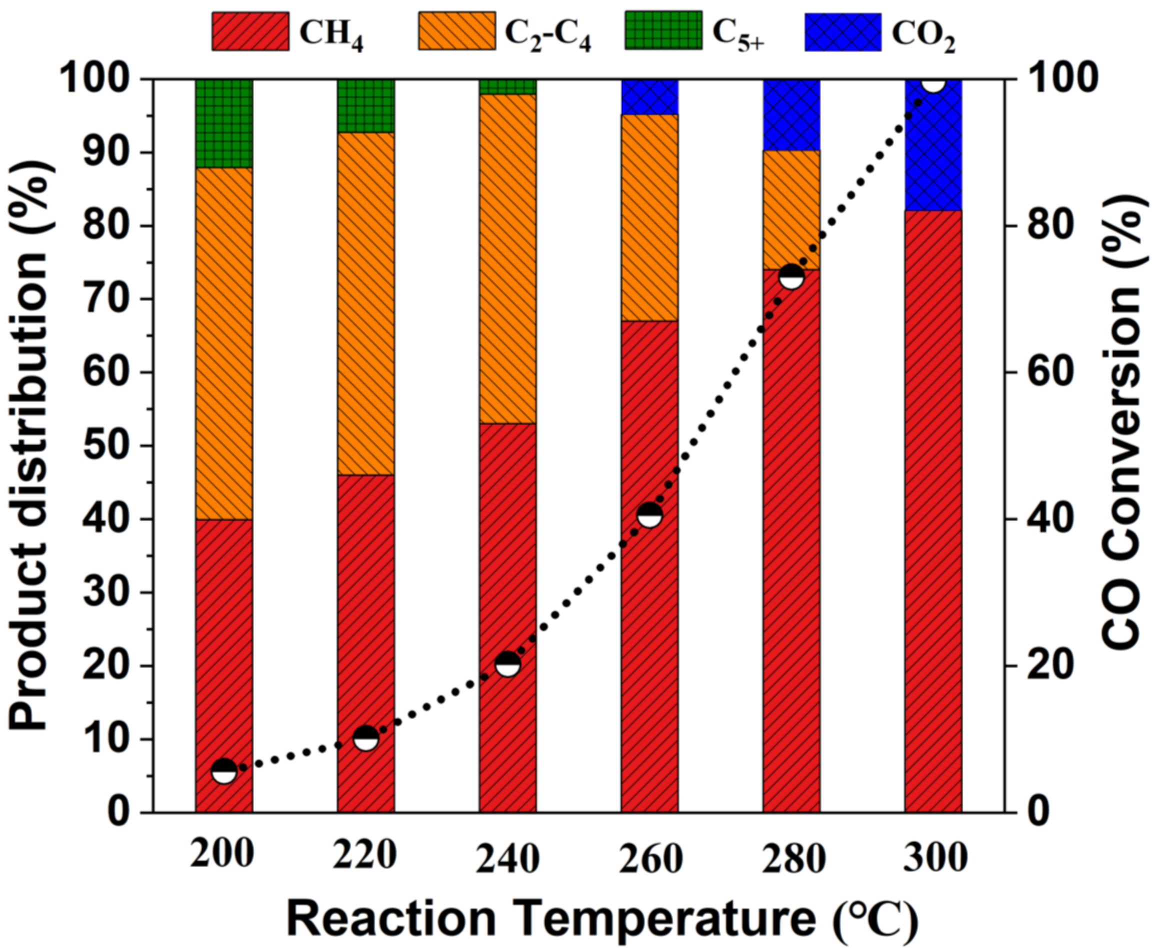

Supplementary Figure 10 | Catalytic performance of CO hydrogenation over 15% Ni/TiO_2 at different temperature. The conversion and product distribution on 15% Ni/TiO_2 at 200, 220, 240, 260, 280, and 300 °C, respectively. Reaction conditions: catalysts (120 mg), 1 bar, syngas (CO:H_2:Ar=32:64:4; 20 mL/min).

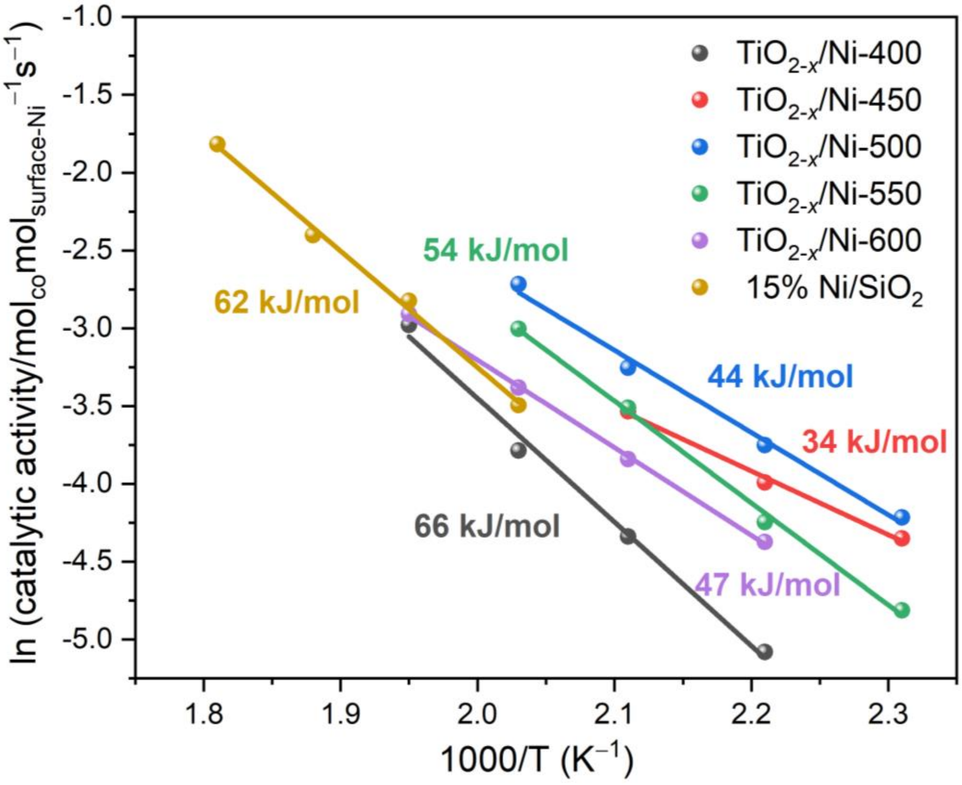

Supplementary Figure 11 | The calculation of activation energy (*E*a). Arrhenius plots for CO hydrogenation over various Ni-based catalysts.

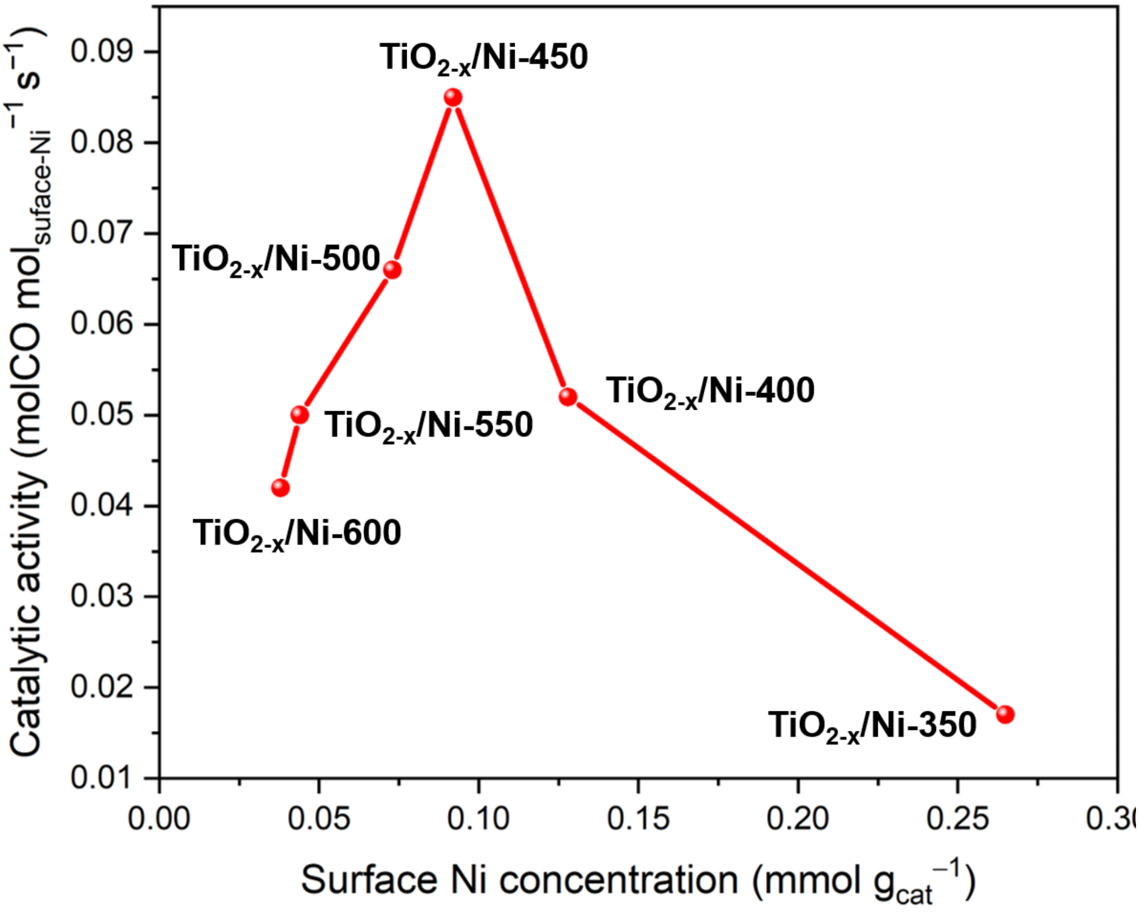

Supplementary Figure 12 | The relationship between catalytic activity and surface Ni concentration. Catalytic activity of CO hydrogenation as a function of surface Ni concentration.

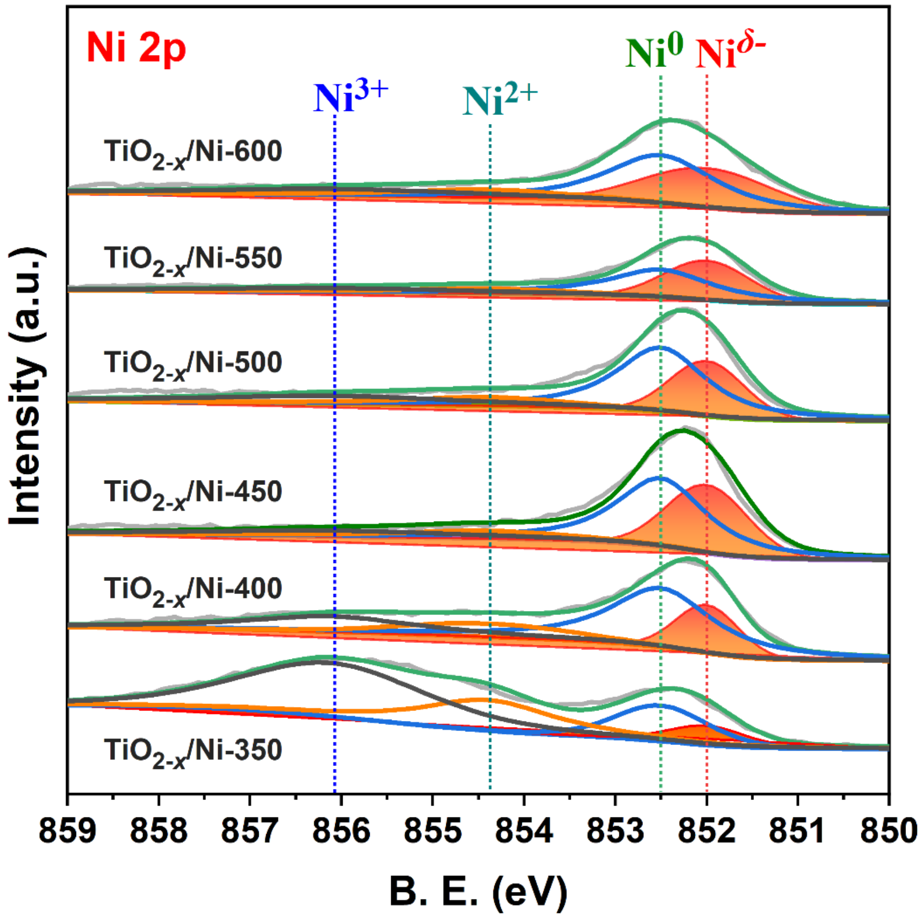

Supplementary Figure 13 | XPS spectra of various TiO_{2-x}/Ni catalysts. XPS Ni2p spectra of various TiO_{2-x}/Ni catalysts with a deconvolution by Gaussian peak fitting method.

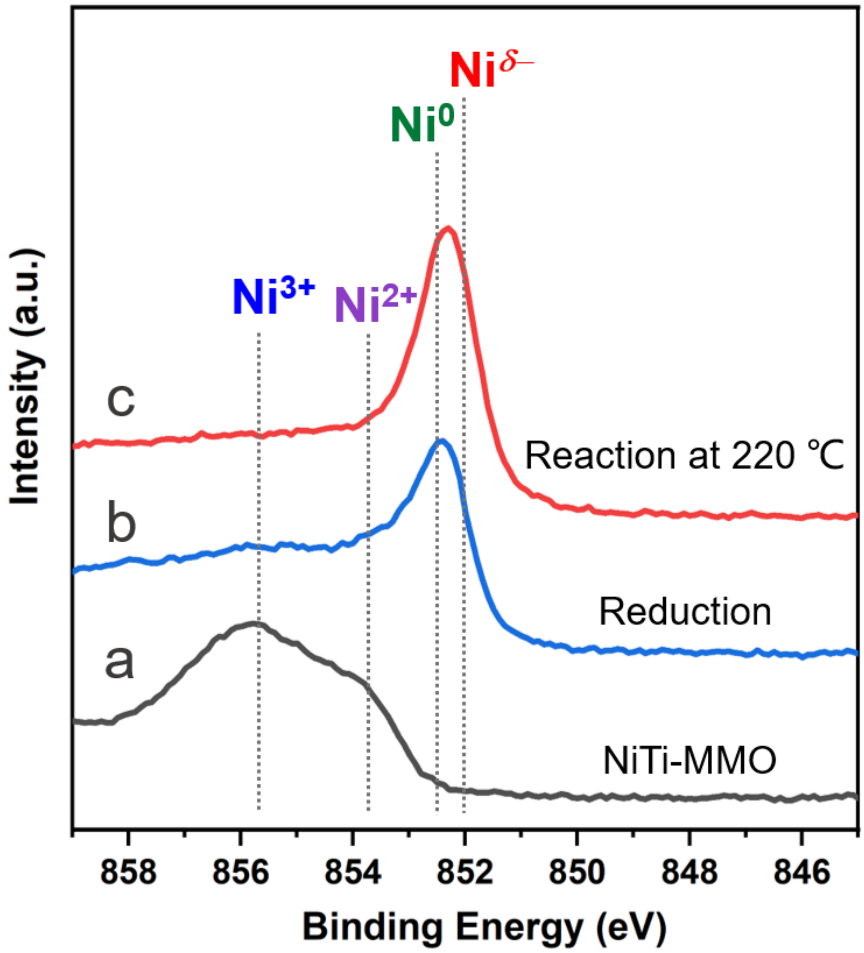

Supplementary Figure 14 | XPS spectra of $TiO_{2\text{-}x}$/Ni-450 catalyst during the reaction. a, NiTi-MMO; **b**, In situ XPS spectra of Ni2p for the $TiO_{2\text{-}x}$/Ni-450 catalyst after the reduction of NiTi-MMO in H_2 atmosphere for 4h; **c**, In situ XPS spectra of Ni2p for $TiO_{2\text{-}x}$/Ni-450 catalyst under the realistic reaction condition.

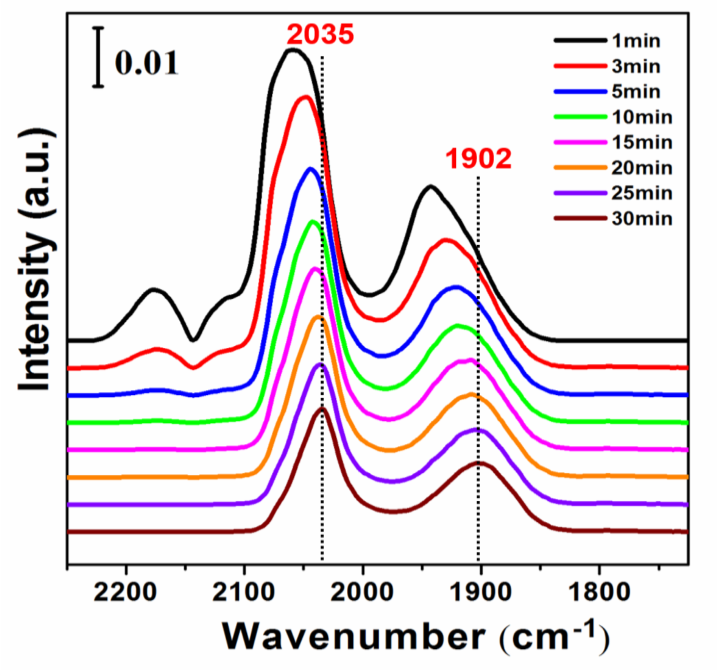

Supplementary Figure 15 | CO-DRIFTS spectra of TiO_{2-x}/Ni-450 catalyst. In situ DRIFTS spectra of CO adsorption on the TiO_{2-x}/Ni-450 catalyst recorded in 2250−1725 cm^{-1} at 250 °C, respectively, after flushing with Ar for 30 min.

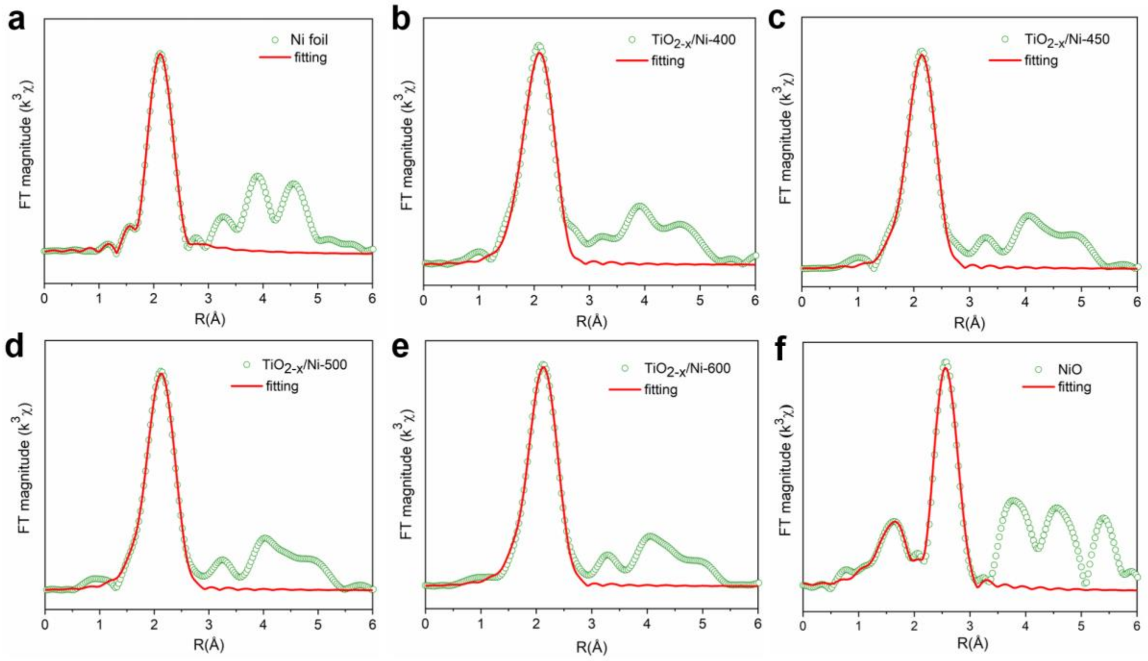

Supplementary Figure 16 | Fourier transform EXAFS spectra and fitting of various TiO_{2-x}/Ni catalysts. Fourier transform EXAFS spectra and fitting at Ni *K*-edge for **a,** Ni foil, **b,** TiO_{2-x}/Ni-400, **c,** TiO_{2-x}/Ni-450, **d,** TiO_{2-x}/Ni-500, **e,** TiO_{2-x}/Ni-600, and **f,** NiO (the dashed line: experimental data; the solid line: fitting curve).

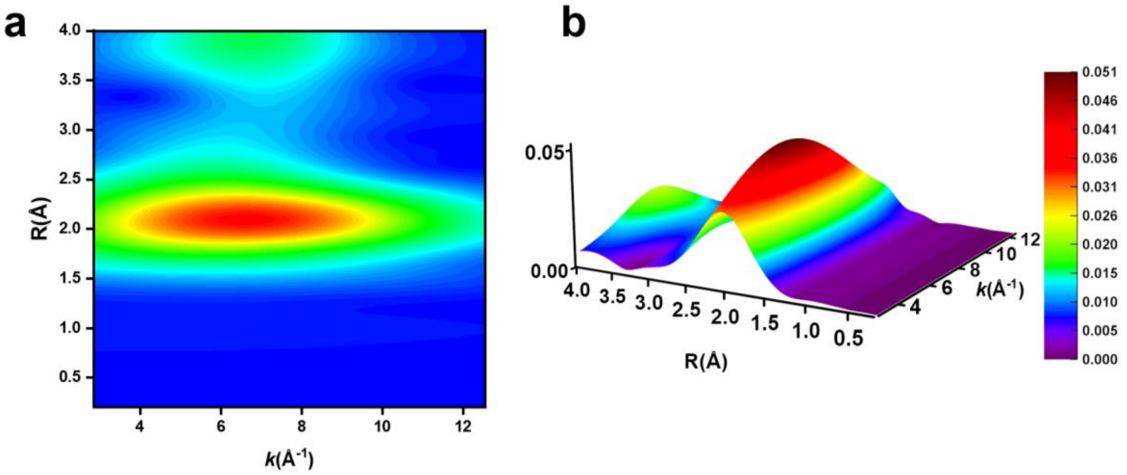

Supplementary Figure 17 | WT analysis. WT analysis of TiO_{2-x}/Ni-400 catalyst in **a** and **b**.

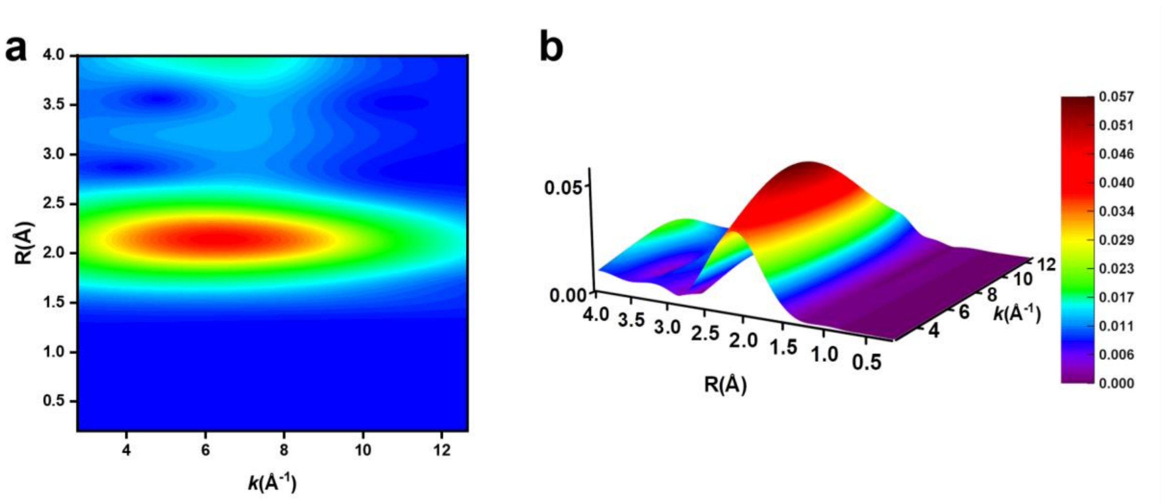

Supplementary Figure 18 | WT analysis. WT analysis of TiO_{2-x}/Ni-500 catalyst in **a** and **b**.

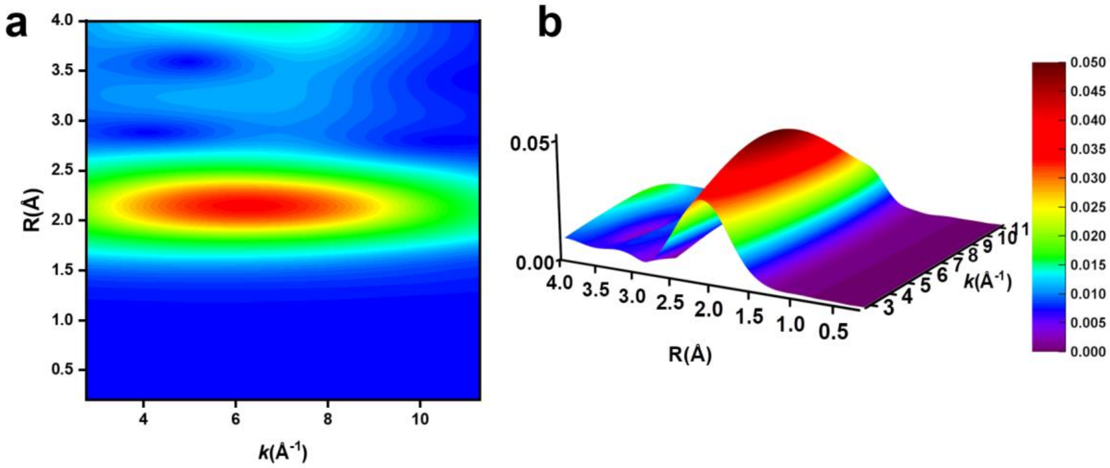

Supplementary Figure 19 | WT analysis. WT analysis of $TiO_{2\text{-}x}$/Ni-600 catalyst in **a** and **b**.

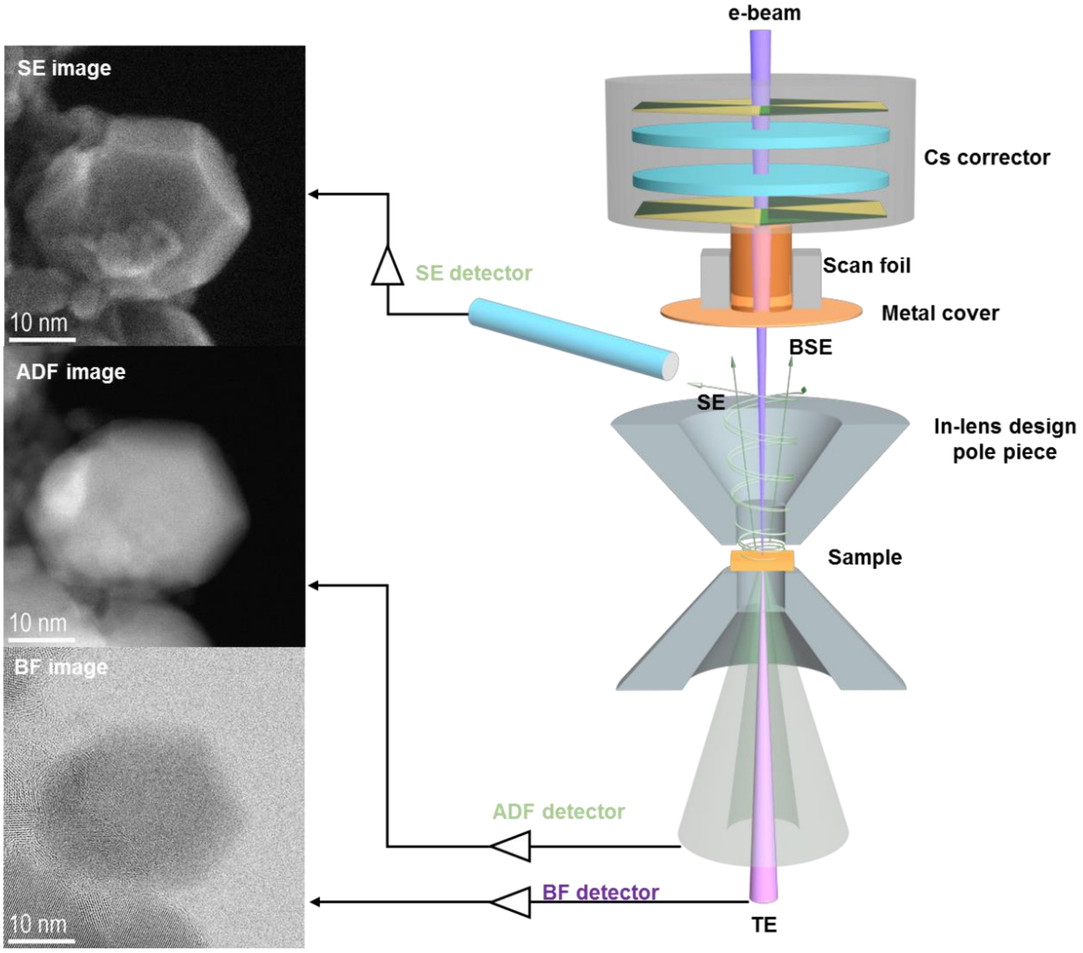

Supplementary Figure 20 | The schematic diagram of *in situ* ESTEM. The schematic diagram of *in situ* ESTEM, which can simultaneously collect the SE image, BF image, and ADF image, respectively.

SE (secondary electron) or BSE (backscattering electron) was used to image morphology and shape of nanomaterial in SEM, which can inspect topography of sample's surface. Recently, S/TEM (scanning transmission electron microscope) has also been equipped with a SE detector to obtain the surface of near-surface information of nanomaterials under STEM mode. A specific advantage of SE mode is: it can successfully visualize the surface of nanomaterials at atomic resolutions regardless of atomic mass and density.

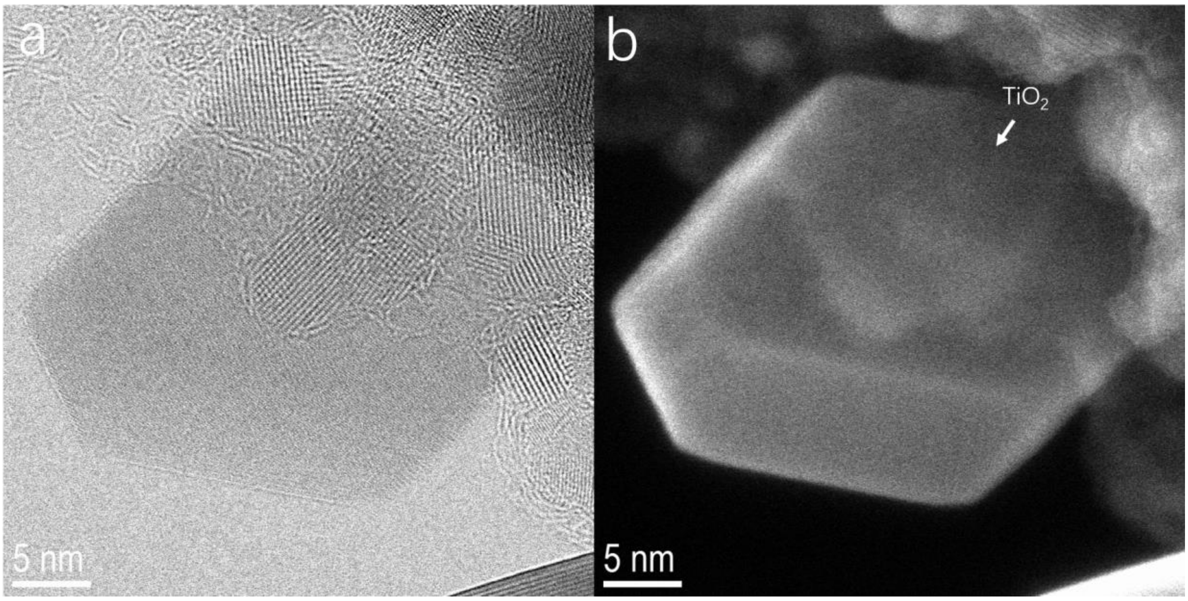

Supplementary Figure 21 | ESTEM images of the $TiO_{2\text{-}x}$/Ni-450 catalyst. *In situ* ESTEM images arising from **a,** BF (backscattering electrons) and **b,** SE (secondary electrons) over the $TiO_{2\text{-}x}$/Ni-450 catalyst after reduction in H_2 atmosphere (10 Pa) at 450 °C, respectively.

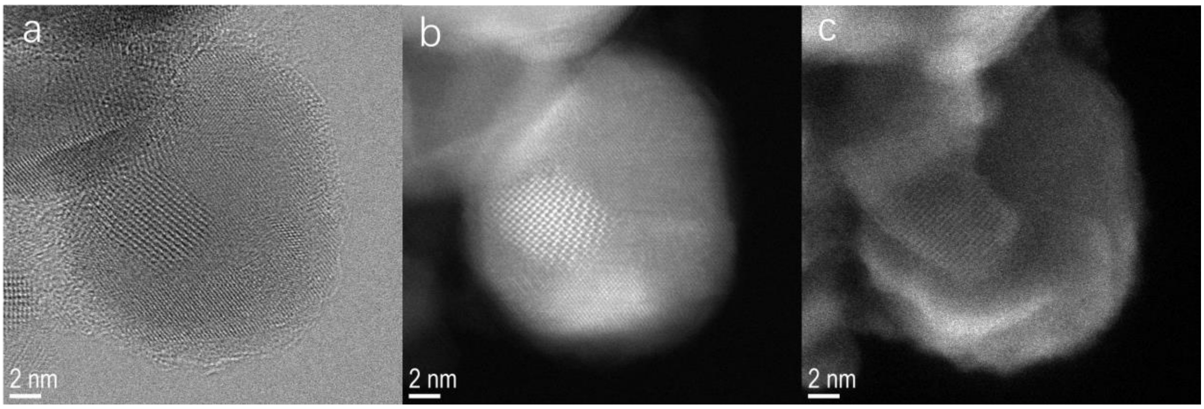

Supplementary Figure 22 | ESTEM images of the TiO_{2-x}/Ni-600 catalyst. ESTEM images arising from **a,** SE (secondary electrons) and **c,** BF (backscattering electrons) as well as collecting **b,** HAADF image over the TiO_{2-x}/Ni-600 catalyst after reduction in H_2 atmosphere (10 Pa) at 600 ℃.

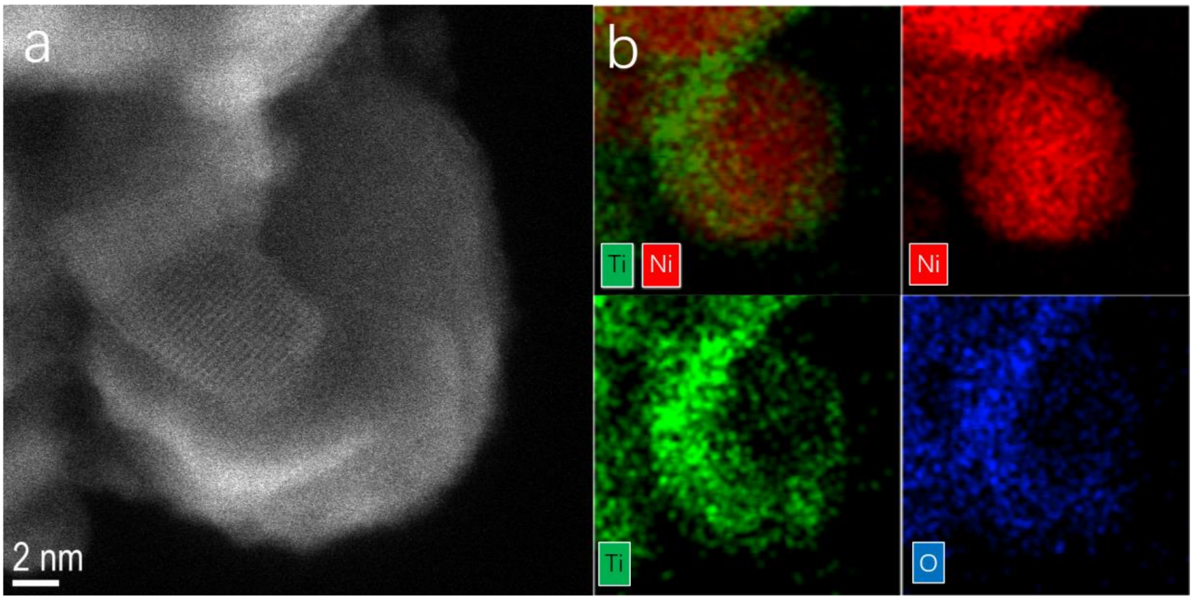

Supplementary Figure 23 | HAADF-STEM image of the TiO_{2-x}/Ni-600 catalyst. a, Ex situ HAADF-STEM image of the TiO_{2-x}/Ni-600 catalyst. **b,** The element EDS mapping of Ni, O, and Ti, respectively, after reduction at 600 °C under H_2 atmosphere.

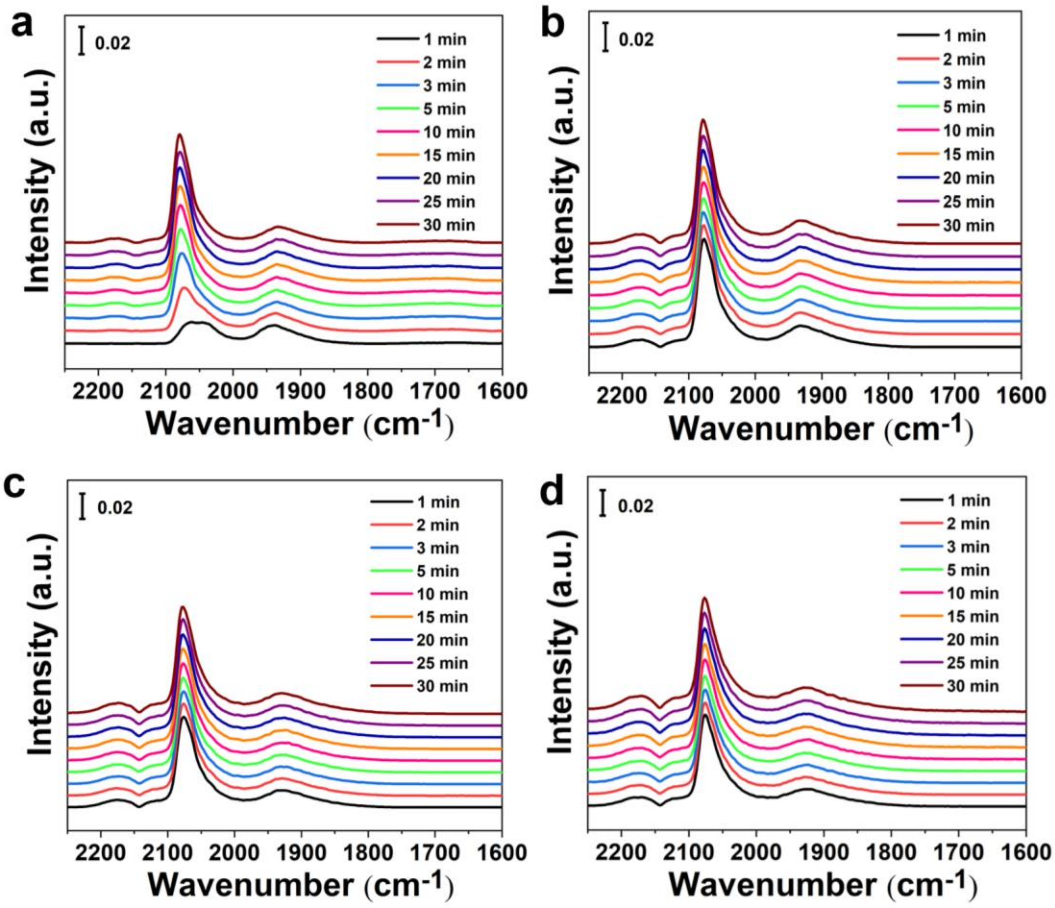

Supplementary Figure 24 | *In situ* CO time-resolved DRIFTS spectra. *In situ* CO time-resolved DRIFTS spectra were carried out over the TiO_{2-x}/Ni-450 catalyst at **a,** 160 ℃, **b,** 180 ℃, **c,** 200 ℃ and **d,** 220 ℃, respectively. The corresponding spectra were recorded in 2250−1600 cm^{-1} at different temperature with the time stream on. From bottom to top: upon exposure to 5% CO/Ar (30 ml/min) for 30min at different temperatures. The experiments were conducted following such procedure: the NiTi-MMO precursor was put into the in situ cell and reduced in 5% H_2/Ar atmosphere (60ml/min) at 450 ℃ for 4 h to obtain the optimal TiO_{2-x}/Ni-450 catalyst, and then cooled down to 160 ℃. Subsequently, 5% CO/Ar was switched into this system. The weak IR band at ~2172 cm^{-1} is attributed to the gaseous CO molecules.

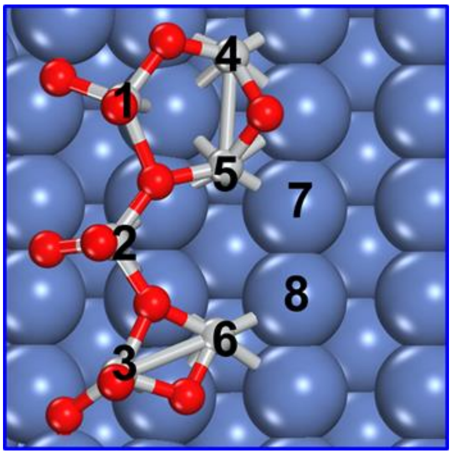

Supplementary Figure 25 | The Bader charge analysis over the Ti_6O_{11}/Ni(110) surface. The optimized structure of Ti_6O_{11}/Ni(110) surface. The Bader charge analysis of labelled atoms is given in **Supplementary Table 7**. The blue, light grey and red spheres are Ni, Ti and O atoms.

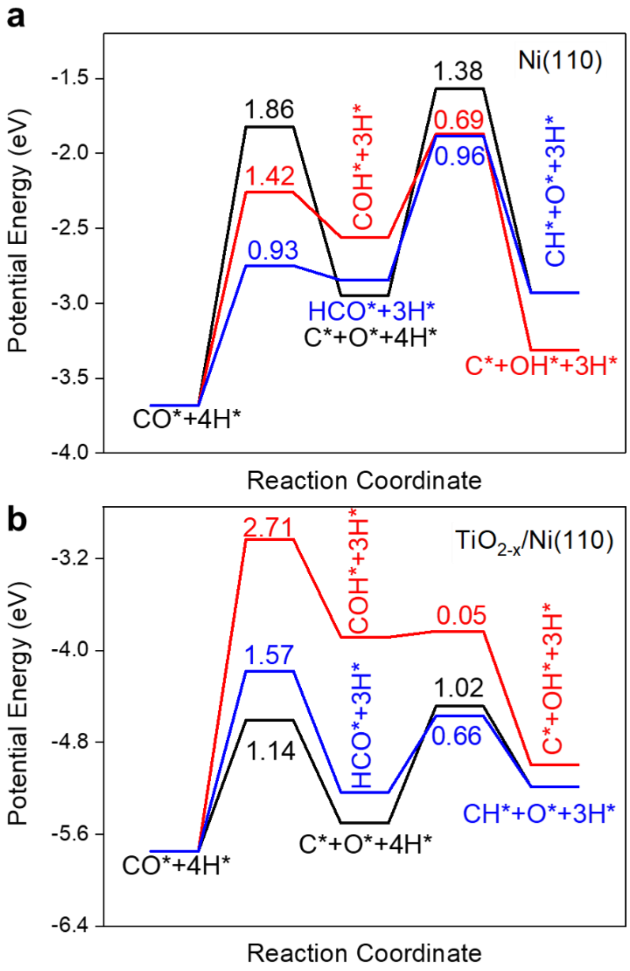

Supplementary Figure 26 | Calculated potential energy diagrams of CO activation. Calculated potential energy diagrams for CO activation in the formation of CH monomer over Ni(110) **(a)** and Ti_6O_{11}/Ni(110) **(b)** surfaces. All the elementary reaction barriers are indicated in eV.

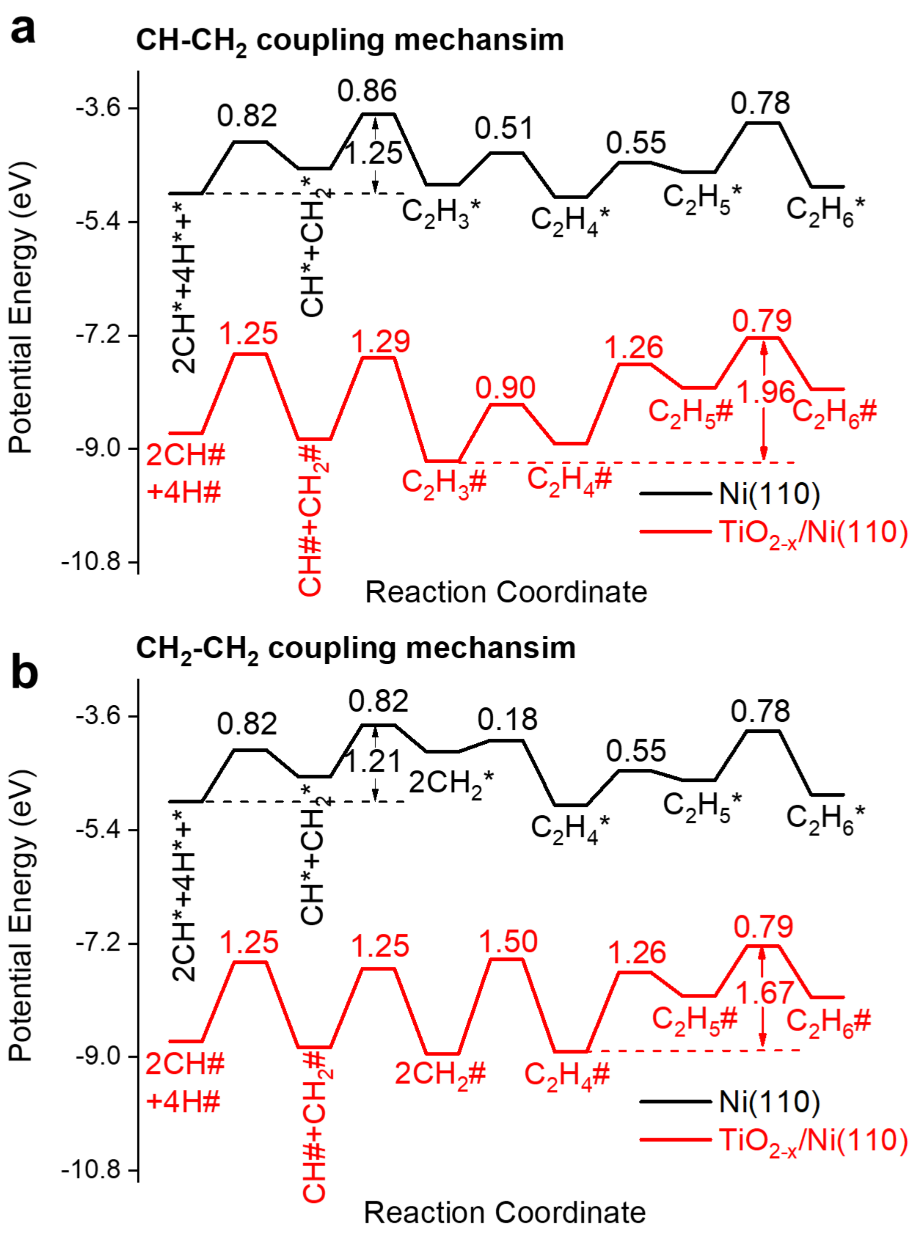

Supplementary Figure 27 | The C_2H_6 formation and coupling mechanisms over Ni(110) and Ti_6O_{11}/Ni(110) surfaces. C_2H_6 formation *via* CH-CH_2 (**a**) and CH_2-CH_2 (**b**) coupling mechanisms over Ni(110) and Ti_6O_{11}/Ni(110) surfaces. All the elementary reaction barriers and the apparent activation barriers are indicated in eV.

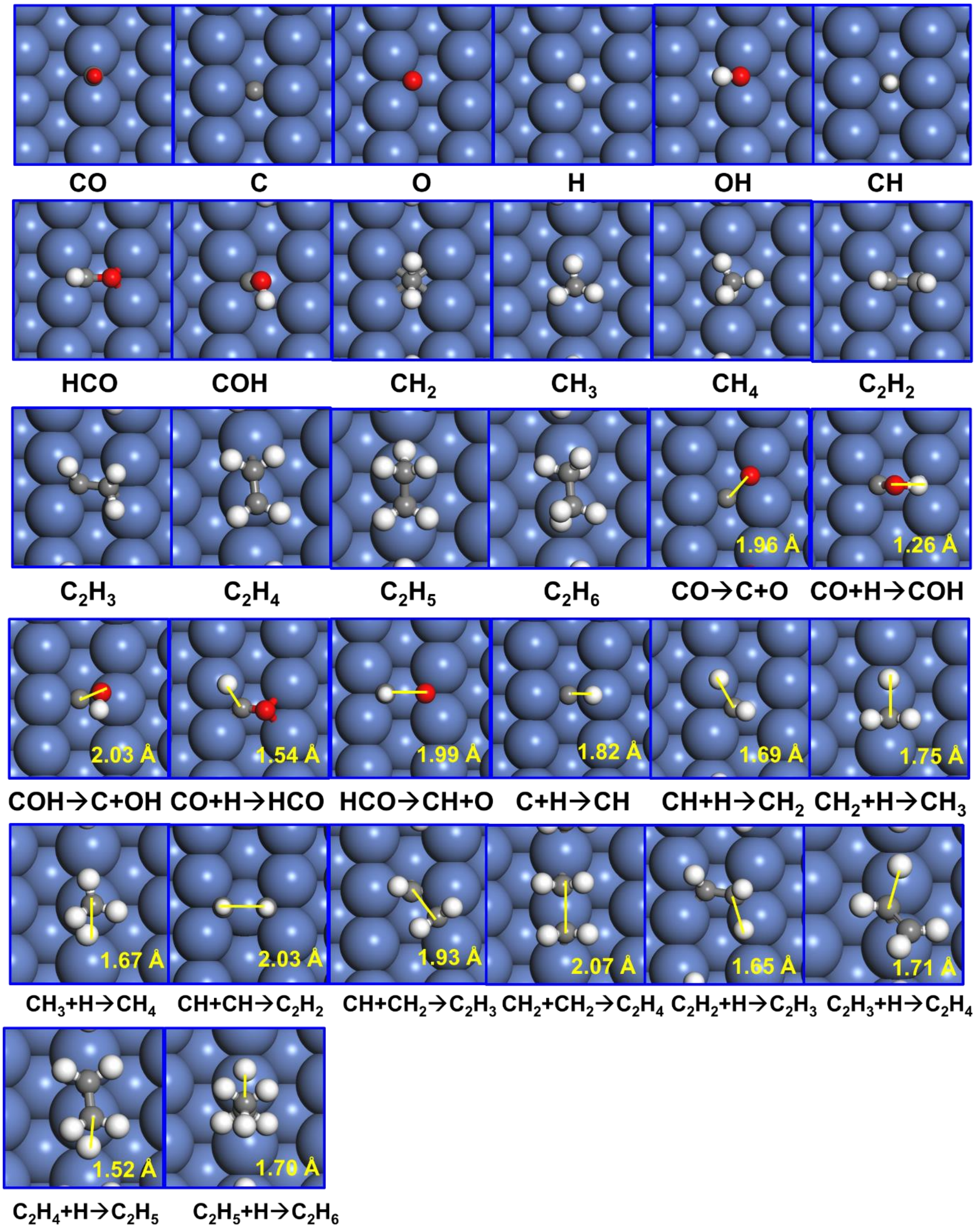

Supplementary Figure 28 | Calculated configurations and the corresponding transition states over Ni(110) surface. Calculated configurations for the adsorption of intermediates and the corresponding transition states in CO hydrogenation towards C_2H_6 over Ni(110) surface. The bond distances at the transition states for CO activation, carbon-carbon coupling and hydrogenation of the intermediates are indicated in Å.

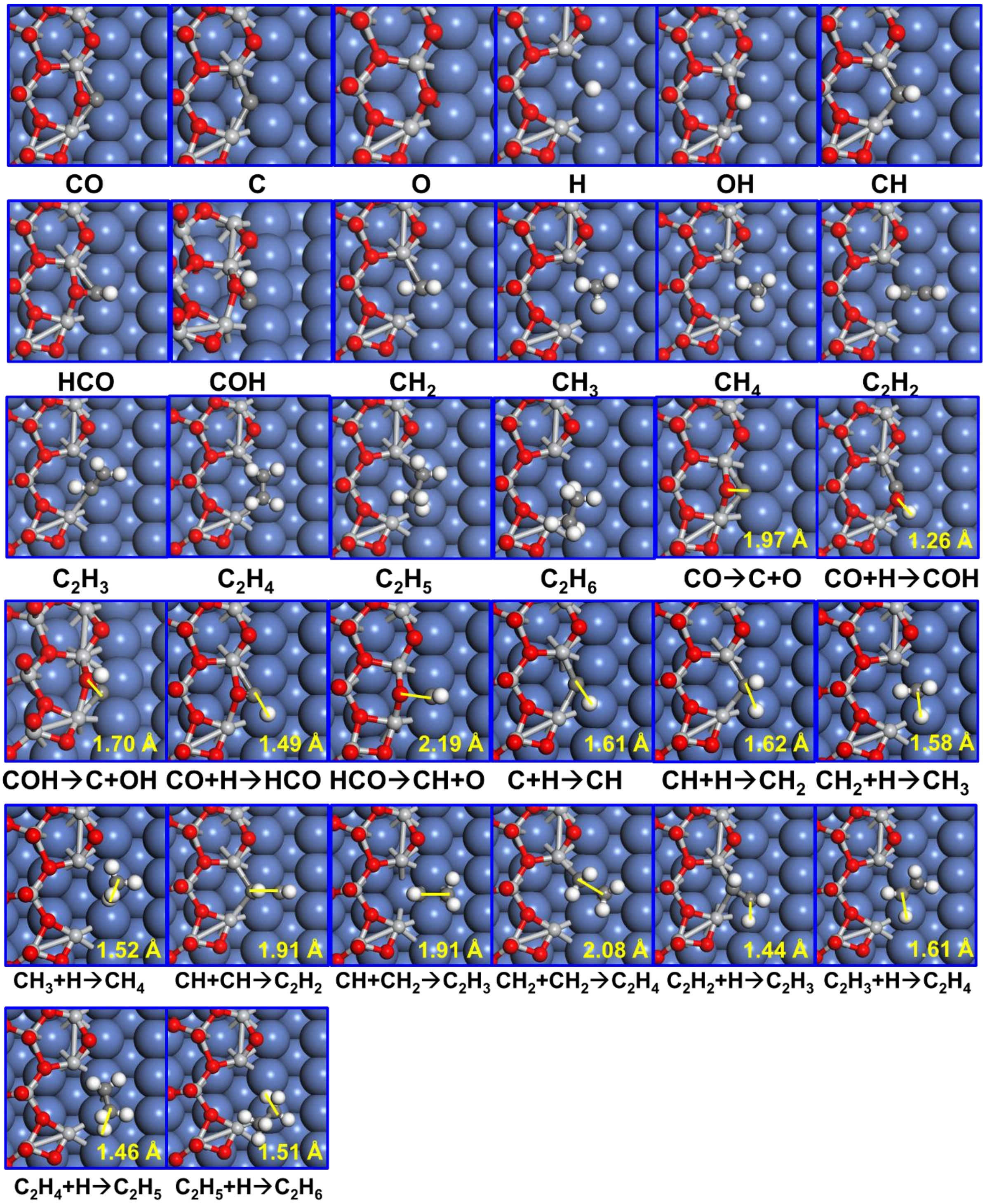

Supplementary Figure 29 | Calculated configurations and the corresponding transition states over Ti_6O_{11}/Ni(110). Calculated configurations for the adsorption of intermediates and the corresponding transition states in CO hydrogenation towards C_2H_6 over Ti_6O_{11}/Ni(110). The bond distances at the transition states for CO activation, carbon-carbon coupling and hydrogenation of the intermediates are indicated in Å.

3. Supplementary Tables

Supplementary Table 1 | Physicochemical properties of various TiO_{2-x}/Ni catalysts.

Sample	Ni species content [a] (wt.%)	Surface Metallic Ni Concentration[b] on ($mmol\ g^{-1}$)	Metal Ni dispersion [c] (%)	X_{CO} [d] (%)	Catalytic activity [e] ($mol_{CO}\ mol_{suface\text{-}Ni}^{-1}\ s^{-1}$)	Reaction rate[f] ($mol_{CO}\ g_{Ni}^{-1}\ h^{-1}$)
TiO_{2-x}/Ni-350	45.5	0.265	3.42	11.5	0.017	0.036
TiO_{2-x}/Ni-400	50.7	0.128	1.48	16.7	0.052	0.047
TiO_{2-x}/Ni-450	53.2	0.092	1.02	19.8	0.085	0.053
TiO_{2-x}/Ni-500	55.4	0.073	0.77	12.1	0.066	0.031
TiO_{2-x}/Ni-550	56.1	0.044	0.46	5.5	0.050	0.014
TiO_{2-x}/Ni-600	56.6	0.038	0.39	3.8	0.042	0.010
15%Ni/SiO_2-450	14.6	0.180	7.20	3.2	0.007	0.031
15%Ni/TiO_2-450	14.7	0.222	8.85	10.1	0.018	0.098

[a] Ni species content was determined by inductively coupled plasma–atomic emission spectroscopy (ICP–AES). [b, c] Surface metallic Ni concentration and metal Ni dispersion were calculated based on the results of CO pulse chemisorption at 200 K (described in Experimental Section). [d] X_{CO} represents the CO conversion of the six various TiO_{2-x}-Ni samples and Ni/SiO_2 at 220 ºC, respectively. [e] The calculated TOF values at 220 ºC. [f] The calculated CO reaction rates.

Supplementary Table 2 | Comparison of the catalytic performance of various TiO_{2-x}/Ni catalysts.

Catalysts	Reaction temperature (℃)	Conversion (%)	Selectivity (%)			
			CO_2	CH_4	C_{2-4}	C_{5+}
TiO_{2-x}/Ni-350	220	11.5	0.1	40.3	43.4	16.2
TiO_{2-x}/Ni-400	220	16.7	0.1	36.0	44.2	19.7
TiO_{2-x}/Ni-450	220	19.8	0.3	35.1	43.1	21.5
TiO_{2-x}/Ni-500	220	12.1	0.5	34.9	43.5	21.1
TiO_{2-x}/Ni-550	220	5.5	0.8	36.4	47.5	15.3
TiO_{2-x}/Ni-600	220	3.8	0.9	37.6	50.2	11.3

Reaction conditions: catalysts (120 mg), 1 bar, syngas ($CO/H_2/Ar$=32/64/4); Space velocity: 10000 mL g_{cat}^{-1} h^{-1}).

Supplementary Table 3 | Comparison of the catalytic performance of TiO_{2-x}/Ni-450 catalysts at different temperature.

Reaction temperature (℃)	Conversion (%)	Selectivity (%)			
		CO_2	CH_4	C_{2-4}	C_{5+}
180	2.1	0.1	18.9	52.6	28.4
200	6.8	0.4	27.3	46.5	25.8
220	19.8	0.3	35.1	43.1	21.5

Reaction conditions: catalysts (120 mg), 1 bar, syngas ($CO/H_2/Ar$=32/64/4); Space velocity: 10000 mL g_{cat}^{-1} h^{-1}).

Supplementary Table 4 | Comparison of the catalytic performance of different Ni-based catalysts.

Catalyst	Reaction pressure (bar)	Temperature (°C)	Selectivity of C_{2+} products (%)	Reaction rate ($mol_{co} g_{Ni}^{-1} h^{-1}$)	Catalytic activity (mol_{CO} $mol_{suface\text{-}Ni}^{-1}$ s^{-1})	Ref
TiO_{2-x}/Ni-450	1	220	64.6	0.053	0.085	This work
15% Ni/SiO_2-450	1	220	32.1	0.031	0.007	This work
15% Ni/TiO_2-450	1	220	54	0.098	0.018	This work
Ni/Nb_2O_5-450	1	230	78	0.058	0.605	11
Ni/*a*-Al_2O_3 -450	1	230	45	0.061	0.008	11
NiCo/Nb_2O_5	1	220	87	0.122	--	12
NiCo/TiO_2	1	220	85	0.050	--	12
NiCo/*a*-Al_2O_3	1	220	60	0.018	--	12

Supplementary Table 5 | Surface Ni species concentration of various TiO_{2-x}/Ni catalysts derived from deconvolution results of XPS.

Sample	Nickel Species Percentage (%)			
	**$Ni^{\delta-}$**	**Ni^{0}**	**Ni^{2+}**	**Ni^{3+}**
TiO_{2-x}/Ni-350	7.2	15.5	25.1	52.2
TiO_{2-x}/Ni-400	16.5	48.8	12.6	22.1
TiO_{2-x}/Ni-450	33.8	53.6	12.6	--
TiO_{2-x}/Ni-500	34.8	51.7	13.4	--
TiO_{2-x}/Ni-550	35.4	50.5	14.0	--
TiO_{2-x}/Ni-600	36.1	50.0	13.9	--

Supplementary Table 6 | EXAFS fitting parameters at the Ni *K*-edge for various TiO_{2-x}/Ni catalysts reduced at different temperatures.

Sample	Shell	*N* [a]	*R* (Å) [b]	σ^2 (Å^2·10^{-3}) [c]	ΔE^0 (eV) [d]	*R* factor (%)
TiO_{2-x}/Ni-400	Ni−O	1.1(±0.6)	2.08(±0.01)	5.0(±0.4)	3.2	0.2
	Ni−Ni (in metallic state Ni)	9.5(±0.6)	2.49(±0.01)	13.9(±0.8)	-3.1	
TiO_{2-x}/Ni-450	Ni−Ni (in metallic state Ni)	10.4(±1.8)	2.48(±0.01)	13.6(±1.3)	5.2	0.7
TiO_{2-x}/Ni-500	Ni−Ni (in metallic state Ni)	10.7(±0.9)	2.48(±0.01)	14.4(±0.7)	4.0	0.8
TiO_{2-x}/Ni-600	Ni−Ni (in metallic state Ni)	10.8(±0.8)	2.48(±0.01)	15.2(±0.7)	4.6	0.6
NiO reference (25 °C)	Ni−O	6.0(±0.3)	2.09(±0.01)	7.6(±0.6)	-3.7	0.4
	Ni−Ni (O-bridged)	11.8(±0.4)	2.95(±0.01)	7.0(±0.4)	-6.3	
Ni foil (25 °C)	Ni−Ni (in metallic state Ni)	11.9(±0.4)	2.48(±0.01)	6.0(±0.3)	-5.8	0.2

[a] *N*: coordination number; [b] *R*: bond distance; [c] σ^2: Debye-Waller factor; [d] ΔE_0: the inner potential correction. *R* factor: goodness of fit. S_0^2, 0.799, was obtained from the experimental EXAFS fitting over Ni foil reference with known crystallographic value, which was then used to all the samples.

Supplementary Table 7 | Bader charge analysis of the labelled i^th atoms (i = 1 – 8) in Ti_6O_{11}/Ni(110) shown in Supplementary Figure 25. The Bader charge of Ti in bulk anatase TiO_2 is also given as a reference.

Atoms	Bader Charge
Ti in Bulk Anatase TiO_2	2.64
Ti1	2.53
Ti2	2.54
Ti3	2.53
Ti4	2.04
Ti5	2.01
Ti6	1.94
Ni7	-0.13
Ni8	-0.28

4. Supplementary References

1. Blöchl, P. E. Projector augmented-wave method. *Phys. Rev. B* **50**, 17953−17979 (1994).

2. Kresse, G., Hafner, J. Ab. initio molecular dynamics for liquid metals. *Phys. Rev. B* **47**, 558−561 (1993).

3. Kresse, G., Furthmüller, J. Efficient iterative schemes for ab initio total-energy calculations using a plane-wave basis set. *Phys. Rev. B* **54**, 11169−86 (1996).

4. Perdew, J. P., Burke, K., Ernzerhof, M. Generalized Gradient Approximation Made Simple. *Phys. Rev. Lett.* **77**, 3865−3868 (1996).

5. Monkhorst, H. J., Pack, J. D. Special Points for Brillouin-Zone Integrations. *Phys. Rev. B* **13**, 5188−5192 (1976).

6. Morgan, B. J., Watson, G. W. A DFT + U description of oxygen vacancies at the TiO_2 rutile (110) surface. *Surf. Sci.* **601**, 5034−5041 (2007).

7. Yan, L., Chen, H. Migration of Holstein Polarons in Anatase TiO_2. *J. Chem. Theory Comput.* **10**, 4995−5001 (2014).

8. Sun, K., Zhao, Y., Su, H.-Y., Li, W.-X. Force reversed method for locating transition states. *Theor. Chem. Acc.* **131**, 1118−1127 (2012).

9. Henkelman, G., Jónsson, H. Improved tangent estimate in the nudged elastic band method for finding minimum energy paths and saddle points. *J. Chem. Phys.* **113**, 9978−9985 (2000).

10. Henkelman, G., Uberuaga, B. P. Jónsson, H. A climbing image nudged elastic band method for finding saddle points and minimum energy paths. *J. Chem. Phys.* **113**, 9901−9904 (2000).

11. Hernández Mejía, C., Vogt, C., Weckhuysen, B. M., de Jong, K. P. Stable niobia-supported nickel catalysts for the hydrogenation of carbon monoxide to hydrocarbons. *Catal. Today* **343**, 56−62 (2020).

12. Hernández Mejía, C., , van der Hoeven, J. E. S., de Jongh, P. E., and de Jong, K. P. , Cobalt−Nickel Nanoparticles Supported on Reducible Oxides as Fischer−Tropsch Catalysts. *ACS Catal.* **10**, 7343−7354 (2020).
